# Millisecond dynamics of an unlabeled amino acid transporter

**DOI:** 10.1038/s41467-020-18811-z

**Published:** 2020-10-06

**Authors:** Tina R. Matin, George R. Heath, Gerard H. M. Huysmans, Olga Boudker, Simon Scheuring

**Affiliations:** 1 Department of Anesthesiology, Weill Cornell Medicine, 1300 York Avenue, New York, NY 10065 USA; 2 Department of Physiology and Biophysics, Weill Cornell Medicine, 1300 York Avenue, New York, NY 10065 USA; 3 Howard Hughes Medical Institute, Weill Cornell Medicine, New York, NY 10065 USA

**Keywords:** Atomic force microscopy, Single-molecule biophysics, Transporters in the nervous system, Atomic force microscopy

## Abstract

Excitatory amino acid transporters (EAATs) are important in many physiological processes and crucial for the removal of excitatory amino acids from the synaptic cleft. Here, we develop and apply high-speed atomic force microscopy line-scanning (HS-AFM-LS) combined with automated state assignment and transition analysis for the determination of transport dynamics of unlabeled membrane-reconstituted Glt_Ph_, a prokaryotic EAAT homologue, with millisecond temporal resolution. We find that Glt_Ph_ transporters can operate much faster than previously reported, with state dwell-times in the 50 ms range, and report the kinetics of an intermediate transport state with height between the outward- and inward-facing states. Transport domains stochastically probe transmembrane motion, and reversible unsuccessful excursions to the intermediate state occur. The presented approach and analysis methodology are generally applicable to study transporter kinetics at system-relevant temporal resolution.

## Abstract (web summary)

Excitatory amino acid transporters (EAATs) are crucial for the removal of excitatory amino acids from the synaptic cleft. Here authors combined high-speed atomic force microscopy line-scanning with automated state assignment for the determination of transport dynamics of Glt_Ph_, a prokaryotic EAAT homologue, with millisecond temporal resolution.

## Introduction

Excitatory amino acid transporters (EAATs) are a family of integral membrane proteins that are essential in the mammalian central nervous system (CNS). This family of proteins assures efficient neurotransmission and prevents glutamate-mediated neurotoxic effects by regulating extracellular transmitter levels of neurons and glial cells surrounding synapses. Through EAA removal from the synaptic cleft, EAATs reset the synapse for the next round of neurotransmission. Malfunction of EAATs results in various neurological pathologies such as poor recovery after stroke and neurodegenerative diseases^1–4^. Expression of glutamate transporters is not exclusive to the CNS, but also occurs in other organs such as kidney (EAAT3) or the retina where EAAT5 mediates glutamate-dependent chloride influx into cells^5–7^. This vital family of transporters symports three sodium (Na^+^) ions and one proton (H^+^) along with the EAA, and antiports one potassium (K^+^) ion^8,9^.

Glt_Ph_ is an archaeal homolog of EAATs from *Pyrococcus horikoshii*. Glt_Ph_ shares ~37% amino acid sequence identity with human EAAT2, and has a high structural similarity with human EAAT1^10^ (RMSD: 1.5 Å) and with a neutral amino acid transporter ASCT2^11^. Glt_Ph_ is an excellent model system to study EAATs: the first structure (outward-facing) was solved in 2004^12^, and since then, about a dozen structures describe various conformational states (outward- and inward-facing, and intermediate) and substrate binding occupancies^13^. Glt_Ph_ is a bowl-shaped homo-trimer comprising a central trimerization domain and three peripheral transport domains (Fig. S1). Each transport domain consists of four transmembrane (TM) helices and two helical hairpins (HP1, HP2) that are essential in substrate binding. HP2 has been shown to play the key role in the gating process on both, the extracellular^14–20^ and the intracellular sides of the membrane^20–23^.

Glt_Ph_, like EAATs, symports three Na^+^-ions (down their electrochemical concentration gradient from the extracellular to the intracellular space), and L-aspartate (Asp, against the concentration gradient) across the membrane. In contrast to EAATs, Glt_Ph_ does not require H^+^-symport or K^+^-antiport to complete the transport cycle^14,22,24^. While other transporters function by the rocker-switch (e.g., LacY^25^) or gated-pore (e.g., LeuT^26^) mechanisms, Glt_Ph_ displays an “elevator” mechanism, whereby the transport domains move ∼1.8 nm across the membrane^12,27–32^. The transport domain movement is a stochastic and thermally driven process, and each protomer is independent of the others within the trimer^19,27,28,30–33^. The “elevator” transport mechanism is general, and prokaryotic and eukaryotic proteins from several families work with “elevator” domains (Table S1, e.g.^3,4,6,34^, for a review see^35^).

Glt_Ph_ transport dynamics have been studied using single-molecule FRET (smFRET) of tethered transporters in detergent^28^ or vesicles^29,30^, and by high-speed atomic force microscopy (HS-AFM) that visualized the dynamics of unlabeled Glt_Ph_ in membrane directly^33^. Although these observations represented tremendous progress in our mechanistic understanding of the transporter, the use of low photon intensity to acquire time-extended FRET traces and the challenge to acquire highly contrasted HS-AFM movies set limits to the spatio-temporal resolution. As a consequence, short-lived events and transport sub-states may have escaped observation.

Here, we develop and apply HS-AFM^36^ line scanning (HS-AFM-LS^37^) on unlabeled molecules and report Glt_Ph_ transport domain movements with unprecedented spatial and temporal resolution. Our results represent the most detailed recordings of individual transport domains under close-to-physiological (at ambient temperature and pressure and in membrane) conditions providing insights into rapid state transitions and transport sub-states.

## Results

### Reconstitution of Glt_Ph_ and HS-AFM imaging

**Fig. 1:**
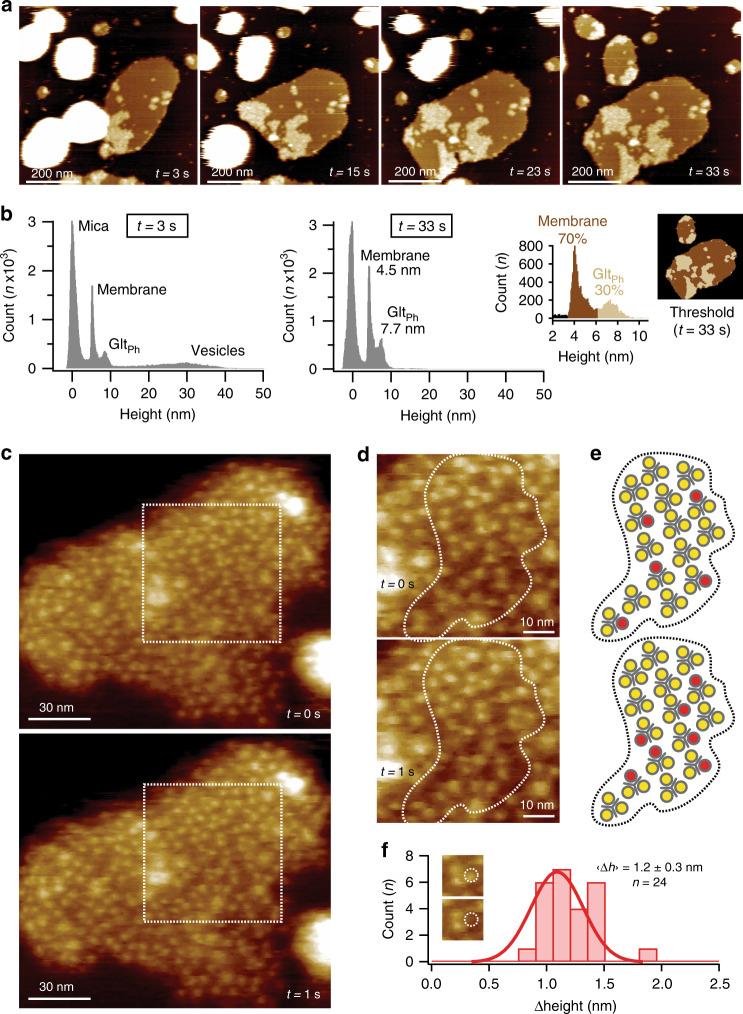
HS-AFM imaging of Glt_Ph_ morphology and activity in membranes. **a** HS-AFM movie frames of Glt_Ph_ proteo-liposomes spreading on mica (Supplementary Movies 1 and 2; representative for >50 experimental replicates). **b** Height distribution analysis: initially (*t* = 3 s) intact vesicles (height: ~30 nm) adsorb to the surface (defined height: ~0 nm) that eventually (*t* = 33 s) spread into membrane sheets (height: ~4.5 nm) with Glt_Ph_ domains (height: ~7.7 nm). Right: membrane packing analysis with thresholds at 3.5 nm < membrane <6.1 nm and 6.1 nm < Glt_Ph_ < 10 nm. Membranes are ~30% Glt_Ph_-packed. **c** Consecutive high-resolution images. **d** Zoomed areas of the dashed outlines in **c** showing Glt_Ph_ trimers and protomer activity (representative for >50 experimental replicates). **e** Schematic representation of the dashed outlines in **d**, where yellow and red circles represent outward- and inward-facing protomers, respectively. **f** Height change (Δheight) distribution of protomer “elevator” motions in **c**. Mean ± s.d. are indicated (*n* = 24). Inset: example protomer (dashed circle).

Purified Glt_Ph_ was reconstituted into vesicles of up to 300 nm in size. The proteo-liposomes were deposited on freshly cleaved mica and imaged in buffer solution. HS-AFM captured proteo-liposomes surface adsorption and spreading (Fig. 1a and Supplementary Movies 1 and 2). Height histogram analysis documented the vesicle adsorption (Fig. 1b, *t* = 3 s), and reported membranes with a thickness of ~4.5 nm containing Glt_Ph_-packed regions with ~7.7 nm thickness after spreading (Fig. 1b, *t* = 33 s). Proteo-liposomes that spread in close proximity fused into larger patches, attesting membrane fluidity (Supplementary Movies 1 and 2). The height difference of ~3.2 nm between Glt_Ph_ domains and bare bilayer resulted in a bimodal height distribution that allowed thresholding and area analysis of the Glt_Ph_-packed domains (Fig. 1b, right): Glt_Ph_ covered only ~30% of the total membrane area. Thus, Glt_Ph_ preferentially arranges in densely packed domains despite the availability of an extended free membrane. Although the dense packing might be a consequence of the chosen lipid composition that is different from the native environment in *Pyrococcus horikoshii*^38^, we note that mammalian EAATs cluster too with up to ~5000 transporters/µm^2^ in glial membranes around synaptic clefts^39,40^.

High-resolution HS-AFM imaging of these domains resolved the Glt_Ph_ trimers and the individual protomers within them well (Fig. 1c, d and Supplementary Movie 3), surpassing in clarity and resolution our earlier HS-AFM data^33^. Comparison with Glt_Ph_ X-ray structures showed that the vast majority of transporters exposed the extracellular face, where the protomers stand out of membrane ~2 nm with an inter-protomer distance of 5.7 nm, to the HS-AFM tip (Fig. S2). Indeed, in the high-resolution images (Fig. 1c, d), the Glt_Ph_-packed domains are covered with molecules protruding ~2 nm leaving essentially no space for molecules inserted in the other direction. In agreement with previous studies^29,33^, only a minority of the transporters displayed dynamics, while the others appeared silent and exposed the transport domains to the extracellular side (Fig. 1e). Dynamic protomers were observed at locations distributed over the entire membrane (Supplementary Movie 3). The range of the “elevator” movement in this experiment was 1.2 ± 0.3 nm, in good agreement with earlier X-ray^10^, HS-AFM^33^, and smFRET^28,30^ data.

### High-speed AFM line scanning (HS-AFM-LS): millisecond transporter dynamics

Among the bulk and single-molecule techniques, HS-AFM stands out with its ability to provide real-time structural and dynamical information of single molecules. HS-AFM images label-free molecules under close-to-physiological conditions with ~0.1 nm vertical and ~1 nm lateral imaging resolution. Furthermore, HS-AFM has typically ~100 ms temporal resolution, giving access to structure–dynamics relationship of proteins^41^, though the achievable imaging speed depends on sample characteristics like scan size and surface corrugation. Recently in a quest to achieve higher temporal resolutions, we used HS-AFM line scanning (HS-AFM-LS^37^) for the analysis of single-protein dynamics. Line scanning, using a conventional AFM, has been used to study protein–protein interactions earlier^42^. In HS-AFM-LS, the slow-scan axis (*y*-direction) is disabled. Therefore, instead of imaging an *x*/*y*-area, we scan over one horizontal *x*-line several hundreds to thousands of times per second, thus reaching millisecond temporal resolution^37^. The topographical readouts of this line are stacked one after another, resulting in kymographs of the dynamical behavior of the molecules. Therefore, HS-AFM-LS has between 2 and 3 orders of magnitude higher temporal resolution than HS-AFM imaging and should allow the detection of fast transporter dynamics and possible intermediate states that have so far escaped kinetic characterization.

**Fig. 2:**
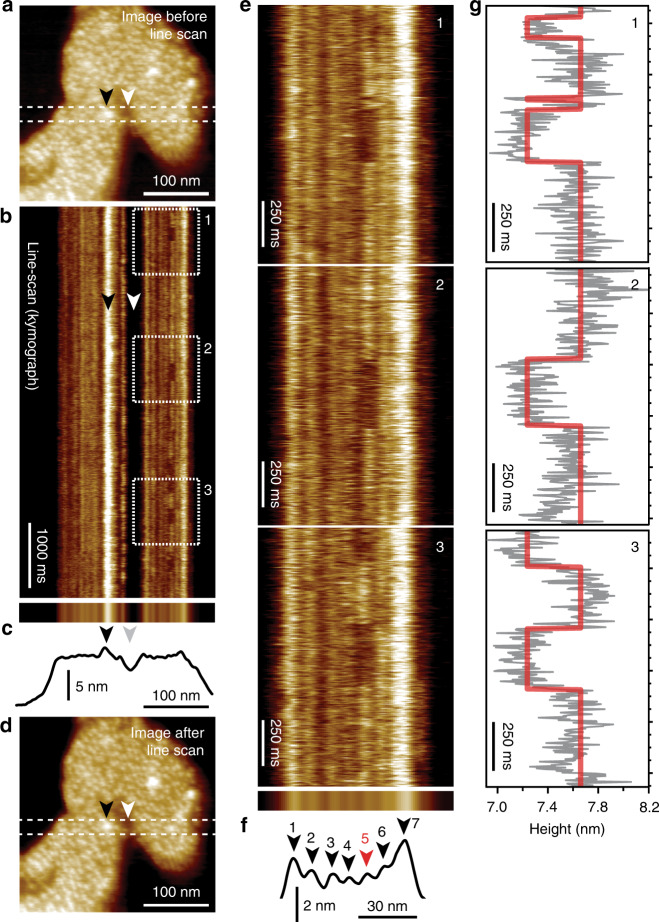
HS-AFM line scanning (HS-AFM-LS): millisecond temporal resolution of unlabeled transporter dynamics. **a** HS-AFM image of a membrane packed with Glt_Ph_ exposing the extracellular face before HS-AFM-LS (apo condition: 20 mM Tris-HCl, pH7.5, 150 mM KCl). Dashed lines indicate the position of the central scan line where subsequent HS-AFM-LS is performed. **b** Six seconds of a HS-AFM-LS kymograph with 3.3 ms line acquisition speed. Each transporter domain appears as a vertical line. **c** Projection (top) and height profile (bottom) of **b**. **d** HS-AFM image after HS-AFM-LS. The lateral position of recognizable features in **a**–**d** are indicated by arrowheads. **e** One second high-magnification views of dashed regions 1, 2, and 3 in **b**. Transport domain excursions to the inward-facing state appear as dark dwells along the vertical time axis. **f** Projection (top) and height profile (bottom) of **e**. Arrowheads indicate the position of the seven protomers in the kymograph (red: active protomer #5). **g** Height/time traces (gray) and state fits (red) of the active domain (protomer #5) in **e**. This figure is representative of the experimental sequence for the >50 replicates analyzed in this work.

HS-AFM-LS experiments begin as imaging experiments to localize a Glt_Ph_ patch and center it in the scan area (Fig. 2a). Disabling the *y*-scan motion engages HS-AFM-LS mode, placing the tip to the central line of the prior imaging frame, and starting repetitive scanning over the protomers located underneath that scan line, thus producing a topography kymograph (Fig. 2b). Projecting the kymograph in the time axis (Fig. 2c, top) allows plotting an average section profile (Fig. 2c, bottom). After recording the kymograph, another area scan demonstrates the absence of lateral stage drift and preservation of the membrane integrity throughout the experiment (Fig. 2d). Close inspection of the kymograph revealed transport domain movements to the inward-facing state as periods of decreased height along the time axis (Fig. 2e). These excursions were in the 250 ms range and shorter and, therefore, would have been missed in area imaging. Projection in the time axis (Fig. 2f, top) and section analysis showed that in this example, seven protomers were tracked but only protomer #5 (Fig. 2f, bottom, red arrowhead) displayed activity during observation (Fig. 2e). To analyze the dynamics of the transport domains, a software to track the *x*-positions (Fig. S3), and a state-transitions and -assignment algorithm to fit the vertical movements (Fig. S4) of the protomers over time were developed and applied, respectively (see “Methods”). In HS-AFM-LS, the single scan line contours transporter domains at varying relative locations of their topographical structure, and therefore the measured “elevator motion” amplitude varies between protomers (Figs. S5 and 2e). We analyzed in detail how stage drift and molecular motion would impact line scan kymographs (Supplementary Movie 4): while X drift leads to positional shift of the protomers in the kymographs, Y drift leads to a change of height, because the scan line crosses the protomer at a different position, i.e. height level. Stage drift varies dramatically, from ~1.0 to 0.02 nm/s, depending on experimental duration and thermal equilibration^37^. Rotation and fast diffusion of trimers lead to loss of their signal in the kymograph. Thus, activity can be determined from traces displaying no drift, or X drift over extended periods, while Y drift leads to decrease of the signal power and loss of the trace. The dense packing of Glt_Ph_ trimers in the membrane typically prevents rotation and diffusion of trimers, and the vast majority of kymographs display height/time traces of protomers that are positionally stable and remain at constant height over tens of seconds. Through the tracking and state assignment approaches, HS-AFM-LS kymographs were transformed into idealized height/time traces that allowed the extraction of transport domain states dwell-times (Fig. 2g).

### HS-AFM-LS detects fast transitions and an intermediate state

To characterize Glt_Ph_ transport-specific dynamics, HS-AFM-LS experiments were performed under relevant environmental conditions, i.e., apo, transport, and inhibitory control conditions. It is, however, important to note that our experimental setup does not allow the establishment of a TM electrochemical gradient. In our experiments, we document the kinetics of the TM passage of either empty (apo) or fully loaded (transport) transporter domains, facing identical conditions on both sides of the membrane.

**Fig. 3:**
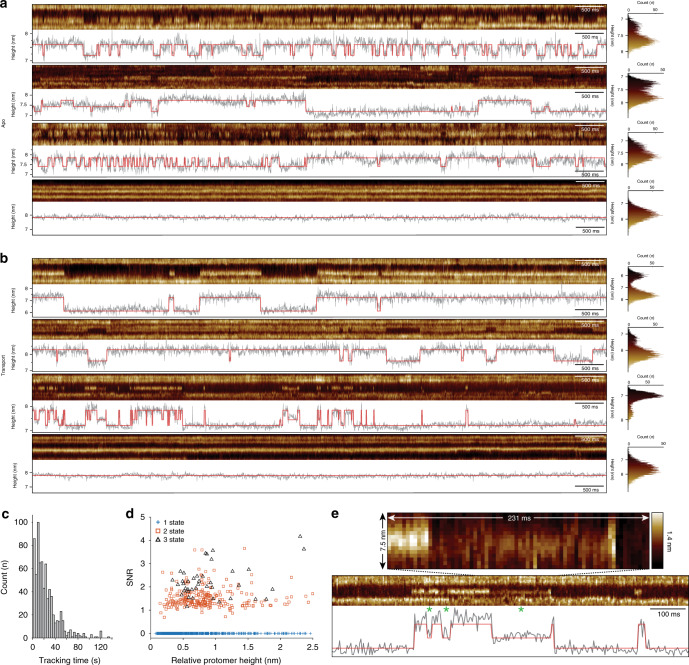
Elevator dynamics of single unlabeled Glt_Ph_ transporter domains. HS-AFM-LS raw data kymographs and height/time traces recorded in apo **a** and transport **b** conditions. All kymographs were recorded at 3.3 ms line acquisition speed. The height/time traces (gray) report the movements of the protomer in the center of the kymograph and are overlaid by the state-transition trace (red). The last kymograph and height/time trace in each condition shows traces of inactive protomers (see Table S2). Right: height distributions of the protomers under investigation. **c** Length distribution of GltPh transport domain tracking in HS-AFM-LS experiments (*n* = 762). **d** Signal-to-noise ratio (SNR) plotted against the protomer relative height over membrane in the outward-facing state for all analyzed 762 individual protomers. Traces assigned with 1, 2, and 3 amplitude states are represented by blue crosses, red squares, and black triangles, respectively. **e** A representative raw data kymograph and height/time trace (gray) overlaid by the idealized state-transition trace (red) of a transport domain visiting three states. Asterisks indicate intermediate state intervals. Top: pixel-by-pixel display of 7.5 nm lateral dimension and 231 ms raw data of the center (dashed lines) of the 3-state kymograph shown below, allowing the viewer to observe the SNR and pixel-statistics of HS-AFM-LS. The full false color scale of the pixels shown is 1.4 nm.

In the absence of substrate, apo condition, Glt_Ph_ was visibly more dynamic and underwent shorter-lived excursions (Fig. 3a), compared to the protein in transport conditions, i.e. in the presence of saturating Na^+^ and Asp concentrations (Fig. 3b). In all conditions, many protomers appeared inactive with their transport domains exposed to the extracellular face (Fig. 3a, b, bottom; see also Fig. 1c). Indeed, only 55% and 23% of protomers of the total of 340 and 422 protomers tracked in the kymographs in apo and transport conditions, respectively, displayed dynamics during our observation window, in agreement with previous work^29,33^. In some previous smFRET works transporters interchanged between extended rests in the outward-facing state and bursts of activity^28–30,33^. Here, we rather observe either inactive or active protomers. We attribute the fact that we do not see an interchange of activity modes to the experimental differences as compared to the mentioned smFRET studies. We hypothesize that conformational sub-states of HP2, specific lipid interactions and lipid composition^38^, protein packing, or membrane deformation effects^43^ underlie the different behaviors of individual protomers in our experiments.

Dynamic protomers were characterized by broad and multimodal height distributions. In contrast, the height distributions of non-dynamic protomers were more uniform (Fig. 3, right column). The protomers’ heights above membrane and motion amplitude were >1 nm in agreement with the transporter domain membrane protrusion height on the extracellular side in the X-ray structures. Glt_Ph_ HS-AFM-LS height/time traces recorded in saturating Na^+^ conditions or in the presence of the inhibitor TBOA display little molecular activity, as expected and in agreement with earlier smFRET and HS-AFM studies^28,33^ (Fig. S6 and Table S2). The HS-AFM-LS traces reached 2 min recording length at 3.3 ms line acquisition speed, and the mean protomer tracking duration was ~23 s (Fig. 3c), which is mainly limited by experiment-dependent stage drift (Fig. S7). At 3.3 ms per scan line, we miss transporter activities shorter than 6.6 ms. Reducing the length of the line scan in the HS-AFM-LS experiments will allow to reach ~1 ms temporal resolution in future studies.

Because HS-AFM-LS contours individual protomers at different relative topographical locations (Fig. S5), each protomer had a different protrusion height above the membrane. Therefore, the height differences between elevator motion states were also different, which, together with the experimental noise, allowed assignment of a signal-to-noise ratio (SNR, see “Methods”) to each trace (Fig. 3d). Note, the experimental height noise in HS-AFM-LS traces on a bare bilayer is Gaussian and has a sub-Angstrom standard deviation (*σ*) at millisecond temporal sampling (Fig. S8). Among the 283 height/time traces of active protomers (apo and transport conditions pooled), the state-assignment algorithm suggested that 40 protomers visited a third state with transporter domain height between the inward- and outward-facing states (Fig. 3d). We term this state “intermediate state” in the remainder of the manuscript, but cannot determine whether this state corresponds to an intermediate state previously observed structurally or computationally^23,44–47^. As expected, the state-assignment algorithm mainly assigned three states in traces with elevated SNR > 2. We tested the performance of the state-assignment algorithm on simulated traces overlaid with varying amounts of noise and found a 93% transition fit accuracy and an 80% state-assignment accuracy at SNR = 2 (Fig. S4). Thus, the intermediate state was assigned, and its kinetics evaluated with confidence. In individual traces with SNR > 2, the intermediate state was directly visible to the eye (Fig. 3e, see also Fig. 3a, b).

Intermediate states have been structurally observed in many transporters^48,49^, including Glt_Ph_^19^ and the closely related Glt_Tk_^23^. However, the kinetics of these states remained unexplored in previous Glt_Ph_ smFRET and HS-AFM data that were interpreted with only the outward- and inward-facing states^33^, with the notable exception of a smFRET study that reported an intermediate FRET-state with a ~1.7 s time constant^30^. HS-AFM-LS combines millisecond temporal resolution with Angstrom vertical resolution (Fig. S8). Thus, HS-AFM-LS surpasses previous approaches in temporal and spatial resolution. Another advantage is that the lateral contouring of each transport domain is reported by several pixels (Fig. 3e, top), and thus, the vertical position is described by several thousands of samples per second, resulting in excellent statistics.

### State dwell-times and transition pathways

**Fig. 4:**
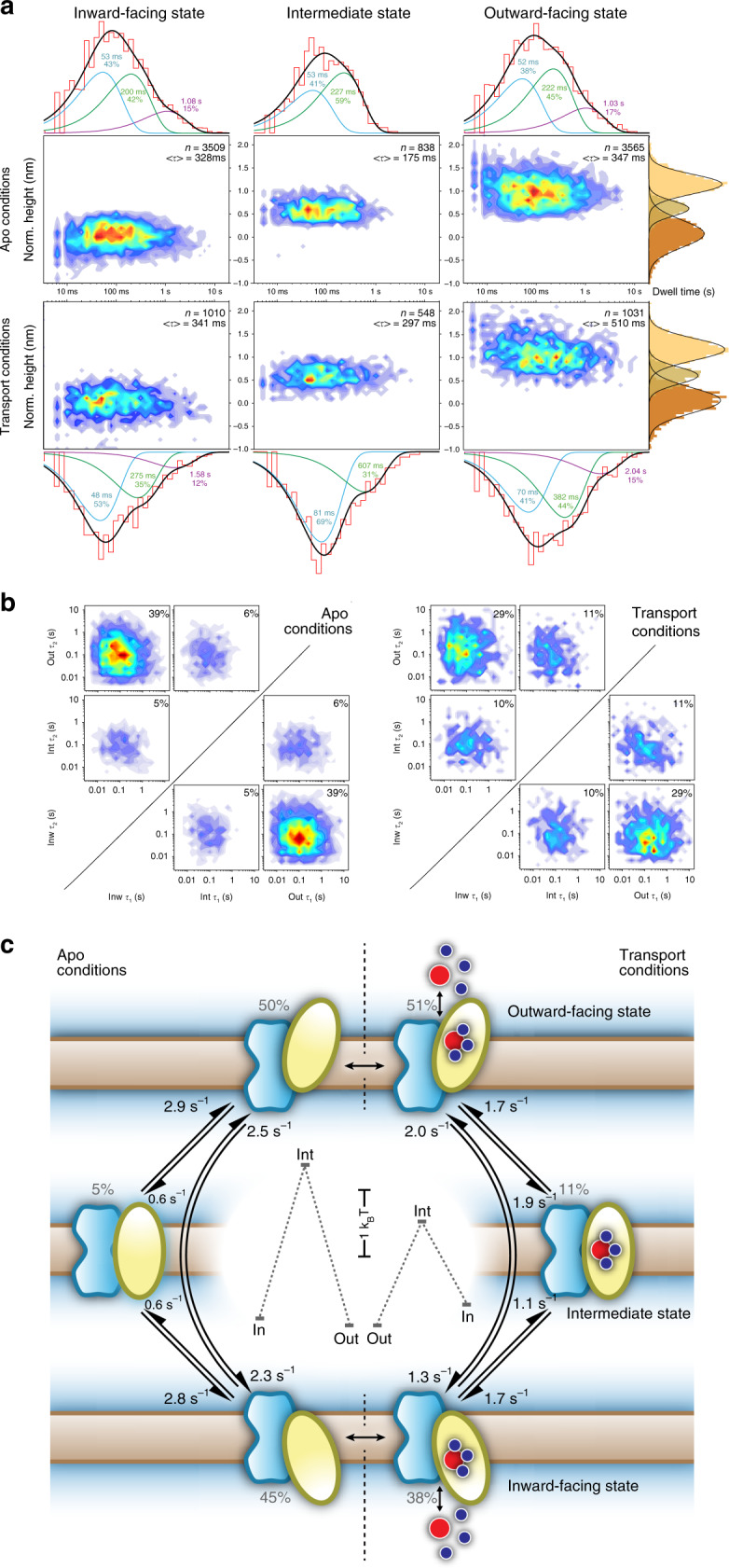
Dynamics of the Glt_Ph_ transport cycle. **a** Density maps of dwell-times vs height of transport domains in the absence and presence of substrates, i.e. apo (top) and transport (bottom) conditions: inward- (left), intermediate (middle), and outward- (right) facing state dwell-times. All density maps and histograms (above and below the density maps) were logarithmically binned. The dwell-time histograms were fitted (black) with multiple exponential components (blue, green, and purple) and the corresponding exponential decay values and occurrence percentages are indicated. The transporter domain height distributions (far right) are well fitted by Gaussians. The total number of transitions (*n*) and the average dwell-times (<τ>) are indicated in the tope right of each panel. **b** State-transition tiles in apo (left) and transport (right) conditions, where the initial state and the final state are plotted on the *x*- and *y*-axis, respectively. Inside the tiles: density maps of the initial- vs the final- dwell-time of each transition. **c** Schematic representation of the Glt_Ph_ transport cycle. HS-AFM-LS characterizes the membrane transitions in either apo- or transport conditions in independent experiments (separated by the black dashed line). Only a single trimerization domain (blue) and transport domain (yellow) are shown. Arrows indicate transport domain pathways. The numbers next to each arrow indicate the weighted rate constants (see “Methods”) from one state to another. The percentages next to each state indicate the mean occupancy (see “Methods”) of each state, from which the energy landscapes (central inset) are calculated (note 1: the energy barriers between states are not determined; note 2: the energy landscape only describes the dynamic protomers). Asp (red dot) and Na^+^ (blue dots) associate and dissociate to/from the transport domain on either side of the membrane.

The Glt_Ph_ single-molecule analysis revealed intricate dynamics with a large variety of molecular behaviors and associated state dwell-times. Earlier smFRET and HS-AFM recordings, at the lower temporal resolution, also reported multiple dwell-time characteristics^29,33^. Here, the dwell-time probability density plots and histograms display multiple kinetic components (Figs. 4a and S9 and Table S2, see “Methods”^50^) in both the absence and presence of substrates. We attribute the differences between our HS-AFM-LS and earlier smFRET dwell-time distributions to the differences in acquisition bandwidth. The millisecond temporal resolution in HS-AFM-LS allows the detection of short excursions, which if missed would lead to longer dwell assignments by concatenating the two neighboring dwells. In apo conditions, protomers spent on average a similar amount of time in the outward- (347 ms) and inward- (328 ms) facing states, while the intermediate state was shorter-lived (175 ms). In the presence of substrates, the protomers were overall less active and spent longer times in the outward- (510 ms), inward- (341 ms), and intermediate (297 ms) states. In both conditions, the outward- and the inward-facing states dwell-time distributions were best fitted with three components, while the intermediate state displayed simpler dwell-time distributions, fitted with two components. The multiple exponential components of the outward- and inward-facing states likely represent a collection of conformations. For example, it has been shown that the HP2 gate must swing away from the transport domain for substrate (un)loading^15,51^. Thus, it can be hypothesized that the outward- and inward-facing states encompass conformational heterogeneity of HP2 and that the longer dwell components comprise such open or otherwise restructured gate states. Thus, unlike the outward-facing state, which has a ~2 s dwell-time component in transport conditions, the intermediate state is always comparatively short-lived in both apo and transport conditions. The intermediate state was, however, on average, significantly longer-lived (297 ms vs 175 ms), and was captured about two times more often (548/2589 = 21% vs 838/7912 = 11%) in the presence, compared to the absence of the substrates. Functionally, an intermediate state must be visited in each TM passage^51^. It is, however, not necessary that the transport domains stall in any intermediate position with a measurable dwell-time. Thus, the comparative shortness and rareness of the observed intermediate states in apo conditions suggest that most of the time, the transport domain undergoes rapid diffusive motions across the membrane. Longer dwell-times and increased observation frequency under transport conditions indicate that the loaded domain has a higher propensity to get stuck during the “elevator” movement; potentially in agreement with a recent cryo-EM study where an intermediate-outward state was found in presence of transport substrates^23^. Longer-lived intermediate states might comprise states where the transport domain laterally dissociates from the trimerization domain^52^. Overall, dynamic protomers showed TM cycling activity with average time constants of 1.4 s^−1^ in apo and 1.05 s^−1^ in transport conditions.

HS-AFM-LS height/time traces allow us to follow the sequence of movements of single protomers for extended periods and provides detailed information on the transport domain state occupancy (Table S2) and pathway probabilities (Table S3). In both the absence and presence of substrates, the transport domains most frequently ventured directly between the outward- and inward-facing states, 78% and 58%, respectively (Fig. 4b). The increased spatio-temporal resolution of HS-AFM-LS revealed furthermore that 22% and 42% of the protomer excursions went to or came from the intermediate state in the absence and presence of substrates, respectively.

The conditional distributions of the consecutive dwell-times of the initial and the final states can be plotted within each state transition tile (Fig. 4b). In apo conditions, these plots show no correlation between consecutive dwell-times. The dwell-time distributions are almost symmetric between tiles of reversed-order transitions, such as from the outward- to the inward- and from the inward- to the outward-facing states, proving that the HS-AFM imaging does not bias the transitions. Also, the outward-to-inward and inward-to-outward tiles appear almost symmetric intrinsically, because, in apo condition, the dwell-time distributions of the outward- and inward-facing states are very similar. In contrast, in transport conditions, the increased outward-facing dwell-times result in asymmetric distributions within the consecutive dwell-time tiles (Fig. 4b, right).

The extended height/time traces allow tracking protomers over several transition steps, providing further insights into the intermediate state kinetics. Specifically, we can distinguish between the situations when the intermediate state is visited measurably during the successful transitions between the outward- and inward-facing states and when it is visited during the failed attempts followed by a return to the starting state (Table S3). This analysis shows that mean dwell-times are pathway-specific. This property is particularly pronounced in transport conditions, but is also detected in apo conditions. We found that for the failed attempts, i.e., out → int → out or in → int → in, the initial mean state dwell-time was about two times longer than when the protomers crossed the membrane, i.e., out → int → in or in → int → out. Thermodynamically, the reverse movements of the failed excursions are expected. However, failed excursions (out → int → out or in → int → in) occur about two times more often than complete transitions (out → int → in or in → int → out). Moreover, the dwell-times of the starting states are longer for the failed compared to complete transitions (Table S3). These observations indicate a structural coupling of the outward- and inward-facing states with their neighboring intermediate state (Table S3, graphical summary).

The establishment of a detailed kinetic model comprising all kinetic sub-states (Fig. 4a) and the pathway-specific state dwell-times and pathway probabilities (Table S3) is beyond the scope of this work, and would need many more hours of recording (we currently recorded 100 and 153 min of protomer kymographs in apo and transport conditions, respectively; Table S2). Instead, we can draw a simplified kinetic transport model with weighted rate constants (see “Methods”) that recapitulates that the transitions between the outward- and inward-facing states can occur by multiple pathways (Fig. 4c). The corresponding energy landscapes highlight that the probability to transition through the intermediate states is higher in transport- compared to apo conditions (Fig. 4c, inset). The energy landscape further illustrates that the outward-facing state is favored in both apo and transport conditions, in agreement with our earlier HS-AFM imaging analysis. In addition, in control experiments with TBOA or saturating Na^+^, protomers were found stalled in the outward-facing state (Fig. S6). This is in contrast to a recent cryo-EM study of Glt_Tk_^23^, where in Na^+^-only conditions two of three protomers stalled in the inward-facing state. This discrepancy could be explained by either differences between these very similar proteins or differences related to the experimental environment. It is, however, notable that in the same study in transport conditions or in presence of TBOA, preference for the outward-facing state has been found^23^, similar to our findings here. This kinetic model and energy landscape are, however, representing dynamic molecules only. A large fraction of molecules is quiescent in the outward-facing state during our experimental time windows, thus if those molecules would be included, the outward-facing state would be a far lower energy state in the cycle.

## Discussion

While ion channel research uses single-molecule electrophysiology to characterize channel kinetics with single-molecule resolution, there is a paucity of similar approaches in transporter research^53^. smFRET^29^ and HS-AFM imaging^33^ have provided solutions, however with much reduced bandwidth. Here, we introduce HS-AFM-LS^37^ for the investigation of single-transporter dynamics with temporal resolution comparable to single-channel recordings. This development allowed us here to detect the rapid translocation kinetics of the dynamic Glt_Ph_ domains and revealed an intermediate state in the transport cycle. It will also be key for the study of mammalian EAATs that are significantly faster than Glt_Ph_^54^. Based on our HS-AFM-LS data on membrane-embedded Glt_Ph_, we extract kinetics which show that each state of the cycle englobes a family of structural sub-states which show distinct mean dwell-times. Perhaps most remarkably, we show that there are multiple transition pathways between the outward- to the inward-facing states, as computationally proposed^52,55^; establishing a detailed kinetic model comprising multiple pathways and intermediates is a future challenge. Readily, we see that in some of these pathways, an intermediate state is populated for over a hundred milliseconds, while in others, the translocation occurs without delays.

## Methods

### Protein purification

Glt_Ph_ was expressed and purified as previously described^12,27^. Briefly, the protein was expressed in *Escherichia coli* DH10b strain with a C-terminal thrombin cleavage site and 8-His-tag. The isolated crude membranes were solubilized in a buffer containing 20 mM Hepes, pH7.4, 200 mM NaCl, 0.1 mM L-aspartate supplemented with 40 mM n-dodecyl β-D-maltopyranoside (DDM) for 2 h at 4 °C. Solubilized transporters were then applied to immobilized metal affinity resin in the presence of 1 mM DDM. The resin was washed in the same buffer supplemented with 40 mM imidazole, subsequently Glt_Ph_ was eluted in the presence of 250 mM imidazole. The 8-His-tag was removed through thrombin digestion overnight, and the protein was further purified by size-exclusion chromatography in a buffer containing 10 mM Hepes, pH7.4, 100 mM NaCl, 0.1 mM L-aspartate, 0.4 mM DDM. The protein was concentrated to ∼5 mg/ml, flash frozen, and stored at −80 °C. Protein concentration was determined by absorbance at 280 nm using an extinction co-efficient of 57,400 M^−1^ cm^−1^ (per monomer).

### Protein reconstitution

Purified Glt_Ph_ was diluted to a concentration of 1 mg/ml in a buffer containing 10 mM Tris, pH7.4, 100 mM NaCl, 10 mM MgCl_2_ supplemented with 0.05% DDM, and aliquoted into 50-μl samples for reconstitution at various lipid-to-protein ratios (LPRs). Lipids, a 1,2-dioleoyl- sn-glycero-3-phosphocholine (DOPC)/1,2-dioleoyl-sn-glycero- 3-phosphoethanolamine (DOPE)/1,2-dioleoyl-sn-glycero-3-phospho-L-serine (DOPS) 8:1:1 lipid mixture (all lipids from Avanti Polar Lipids), were pre-solubilized in 2% DDM and added at LPRs between 0.5 and 1.0 (w:w). After 1 h of equilibration, ∼5 mg of wet BioBeads (BioRad) were added to each reconstitution trial for detergent removal. After overnight incubation, BioBeads were removed from the sample and the reconstitutions checked by negative-stain electron microscopy for the presence of well-contrasted (protein-packed) vesicles of size sufficiently large (>100 nm) for HS-AFM analysis.

### Sample preparation

A 2-μl drop of the Glt_Ph_-reconstituted vesicles was deposited on a 1.5 mm diameter freshly cleaved mica surface, which was glued with epoxy to a quartz sample stage. After 30 min incubation in a humid chamber, the sample was gently rinsed with imaging buffer and mounted in the HS-AFM fluid cell^33,56,57^.

### High-speed atomic force microscopy (HS-AFM)

All images in this study were taken using a HS-AFM (SS-NEX, RIBM, Tsukuba, Japan)^36^ operated in amplitude modulation mode (with typical free and setpoint amplitudes, *A*_free_ = 1.0 nm and *A*_set_ = 0.9 nm, respectively^58,59^ using optimized scan and feedback parameters. Ultrashort (8 μm) cantilevers (USC-F1.2-k0.15, NanoWorld, Neuchatel, Switzerland) with nominal spring constant of 0.15 N/m, and resonance frequency of ∼650 kHz and quality factor of ∼1.5 in buffer, were used. In the presented experiments, four different buffer conditions were used. No substrate (apo) conditions: 20 mM Tris-HCl, pH7.5, 150 mM KCl; transport conditions (Na^+^ and Asp): 20 mM Tris-HCl, pH7.5, 150 mM NaCl, 1 μM Asp; saturating Na^+^ conditions: 20 mM Tris-HCl, pH7.5, 1 M NaCl; and inhibitory conditions (blocker): 20 mM Tris-HCl, pH7.5, 150 mM NaCl, 1 mM DL-TBOA. We investigated Glt_Ph_ from two protein purifications from which we made ∼5 reconstitutions, at ~5 LPRs (LPRs between 0.5 and 1.0 (w:w)) each.

### High-speed atomic force microscopy line scanning (HS-AFM-LS)

To improve the temporal resolution for the characterization of transporter motions, the spatial dimensionality of data acquisition was reduced^60^. Disabling the slow-scan axis (*y*-direction), the temporal resolution is improved by a factor corresponding to the number of scan lines acquired in the image right before slow-scan axis disabling, typically ~300 times, e.g., from 1 s temporal resolution in HS-AFM movies to 3.3 ms line acquisition speed, i.e. 6.6 ms temporal resolution, in HS-AFM-LS kymographs. Importantly, alternating between HS-AFM image and HS-AFM-LS kymograph acquisition does not change the physics of the data acquisition, in terms of tip oscillation, applied forces, actual tip velocity with respect to the sample, and feedback operation. The noise in HS-AFM-LS traces acquired on pure lipid bilayers of the same composition as the protein is reconstituted in the sub-Angstrom range^60^ (Fig. S8). If anything, HS-AFM-LS operates slightly better than HS-AFM imaging, because imprecisions from scanning the slow-scan axis are eliminated.

### Data analysis

HS-AFM-LS kymographs were contrast adjusted, drift corrected, and assembled by routines and home-written analysis software in Igor Pro Software (WaveMetrics, Lake Oswego, OR, USA) and ImageJ (Fig. S3). To analyze HS-AFM-LS kymographs, (1) each protomer was tracked, (2) its signal transferred into a height/time trace, and (3) analyzed by a state detection algorithm using MATLAB (Matlab, Mathworks, Natick, USA). In brief, a 1D tracking algorithm was applied to the kymographs by low-pass filtering the time axis allowing the automatic detection of each protomer peak position over time. To allow tracking of protomers over extended inward-facing periods a track-linking algorithm is used that “looks forward in time” for the protomer to reappear at its position. The tracking information is then used to read the accurate height/time traces of each protomer from the unfiltered kymograph. It is possible that a protomer is lost through drift and later tracked again—such traces would be counted as two individual protomers. To determine the number of states and state transitions, we adapted the Step Transition and State Identification (STaSI)^61^ algorithm developed for discrete single-molecule data analysis for our HS-AFM-LS data^60^. The STaSI algorithm detects step transitions using Student’s *t* test and then groups segments by hierarchical clustering. The optimum number of states is suggested by weighing between the complexity of the model and the goodness of the fit (minimum description length) to find the simplest model with the least fitting error. We extensively tested the STaSI algorithm by generating traces with two or three states and overlaid them with large amounts of noise (Fig. S4). This provided us with an objective assessment of the state-transition detection and state-number assignment accuracy of the algorithm, because we knew precisely the signal of the simulated trace without noise addition. In addition, the results were compared against another leading single-molecule state detection algorithm (vbFRET). The data showed that the STaSI algorithm outperformed vbFRET for the 3-state assignment particularly in the range of SNR we are dealing with. Both algorithms performed similarly well for the 2-state assignment.

### The signal-to-noise ratio (SNR) of height/time traces

The SNR of each height/time trace is calculated by dividing the height difference between the fitted values of the outward- and inward-facing states Δ*h*_(out-in)_ with the standard deviation of the noise (on each state) in the trace std_(state)_:

1 $${\rm{SNR}} = {\mathrm{{\Delta}}}h_{({\rm{out - in}})}/{\rm{std}}_{({\rm{state}})}.$$

### Dwell-time representation and fitting

For clarity of illustration (Fig. 4a), the dwell-times plots were displayed following the Sigworth method^50^, a standard to display dwell-time distributions of single-channel recordings. Dwell-time distributions were fitted with multi-exponential fits and reduced *χ*^2^ tests were used to avoid overfitting of the dwell-time distributions.

### Kinetic scheme representation

For illustration of a simplified kinetic scheme (Fig. 4c), weighted rate constants *k*′ for each transition were calculated from average dwell-times of each state <*τ*_(state−*n*)_> weighted by the relative occurrence $n_{({\rm{state}} - n \to {\rm{state}} - m)}$ of the transition:

2 $$k^{\prime}_{({\rm{state}} - 1 \to {\rm{state}} - 2)} = 1/\langle\tau _{({\rm{state}} - 1)}\rangle \times n_{({\rm{state}} - 1 \to {\rm{state}} - 2)}/n_{({\rm{state}} - 1 \to {\rm{state}} - 2)} + n_{({\rm{state}} - 1 \to {\rm{state}} - 3)}.$$

To derive the state distribution probabilities (Table S2 and Fig. 4c), the average probabilities $\rho _{({\mathrm{state}} - {{n}})}$of finding an active protomer in a given state was calculated from average dwell-times of each state <*τ*_(state−*n*)_> and the relative occurrence $n_{(state - n)}$ of the state:

3 $$\rho _{({\rm{state}} - 1)} = n_{({\rm{state}} - 1) \times } \langle\tau _{({\rm{state}} - 1)} \rangle /n_{({\rm{state}} - 1)} \times \langle\tau _{({\rm{state}} - 1)} \rangle + n_{({\rm{state}} - 2)} \times \langle\tau _{({\rm{state}} - 2)} \rangle \\ \quad+ n_{({\rm{state}} - 3)} \times \langle\tau _{({\rm{state}} - 3)}\rangle.$$

From the state distribution probabilities (Table S2 and Fig. 4c), the energy differences between states ${\mathrm{{\Delta}}}G_{({\rm{state}} - n \leftrightarrow {\rm{state}} - m)}$ was calculated:

4 $${\mathrm{{\Delta}}}G_{({\rm{state}} - n \leftrightarrow {\rm{state}} - m)} = k_BT \times ln\left( {\rho _{({\rm{state}} - n)}/\rho _{({\rm{state}} - m)}} \right).$$

### Reporting summary

Further information on research design is available in the Nature Research Reporting Summary linked to this article.

## Supplementary information

Supplementary Information

Supplementary Movie 1

Supplementary Movie 2

Supplementary Movie 3

Supplementary Movie 4

Reporting Summary

## Data Availability

Data supporting the findings of this manuscript are available from the corresponding author upon reasonable request. Source data are provided with this paper.

## Source data

Source Data

## Code availability

MATLAB codes used for data analysis are provided in Supplementary Information file Supplementary MATLAB Codes.
