# Supplementary material for: Millisecond dynamics of an unlabeled amino acid transporter
Source: Nat Commun. 2020 Oct 6;11:5016. doi: 10.1038/s41467-020-18811-z (PMC7538599; doi:10.1038/s41467-020-18811-z)
Supplement: Supplementary file 1 — Supplementary Information [file 41467_2020_18811_MOESM1_ESM.docx]

**Millisecond dynamics of an unlabeled amino acid transporter**

Tina R Matin^1,2,#^, George R Heath^1,2,#^ , Gerard HM Huysmans^2^, Olga Boudker^2,3^ and Simon Scheuring^1,2,^*

^1^ Weill Cornell Medicine, Department of Anesthesiology, 1300 York Avenue, New York, NY-10065, USA.

^2^ Weill Cornell Medicine, Department of Physiology and Biophysics, 1300 York Avenue, New York, NY-10065, USA.

^3^ Howard Hughes Medical Institute, Weill Cornell Medicine, New York, NY-10065, USA.

^#^ Equal author contribution

* Correspondence to: [sis2019@med.cornell.edu](mailto:sis2019@med.cornell.edu)

**Supplementary Information**

**Supplementary Figure S1**

| **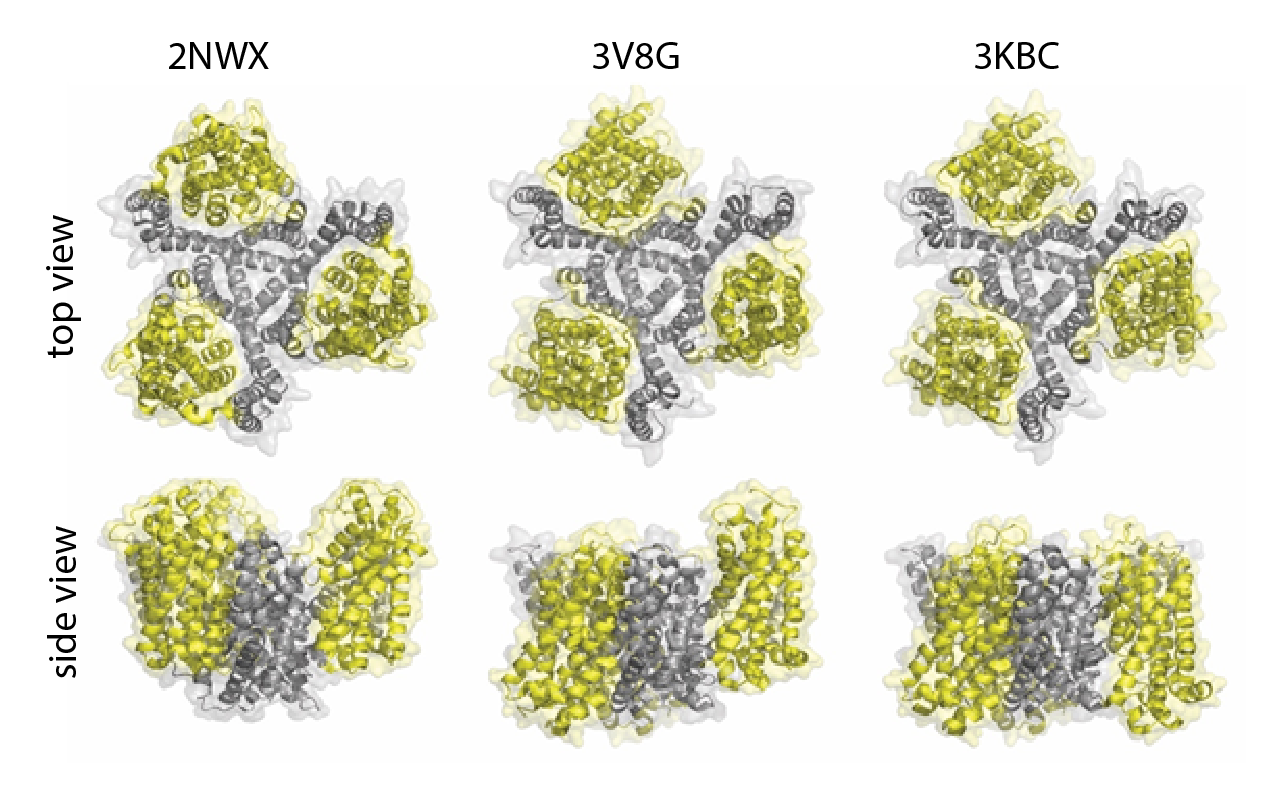** |
| --- |
| **Supplementary Figure S1) Glt_Ph_ X-ray structures in various conformational states. a)** Top views (top row) and side views (bottom row) of Glt_Ph_ high-resolution structures in the outward- (left, PDB 2NWX), an intermediate (middle, PDB 3VBG) and the inward- (right, PDB 3KBC) facing states. The trimerization domain is shown in grey and the transport domains are shown in yellow. Structure alignment on the trimerization domain and preparation of the panels was performed in PyMol. |

**Supplementary Figure S2**

| 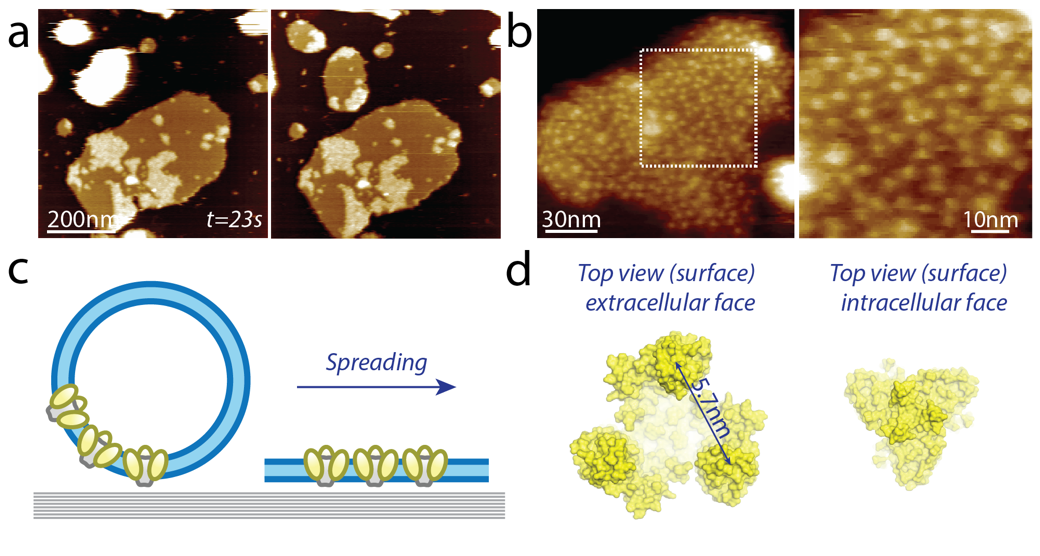 |
| --- |
| **Supplementary Figure S2) Sidedness assignment of Glt_Ph_ in the reconstituted vesicles. a)** HS-AFM movie frames (Supplementary Movie 1, see also Figure 1) of vesicles with reconstituted Glt_Ph_ spreading on the mica support. **b)** High-resolution images of Glt_Ph_ (left) and zoomed image of the dashed outline (right). Panels (a) and (b) are representative for >50 experimental replicates. **c)** Schematic representation of reconstituted Glt_Ph_ in a liposome before and after spreading on the mica support. **d)** Extracellular and intracellular views (molecular surface representation) of the Glt_Ph_ structure in the outward-facing state (PDB 2NWX), allowing unambiguous assignment of the trimer topographies in AFM to the extracellular face. |

**Supplementary Figure S3**

| **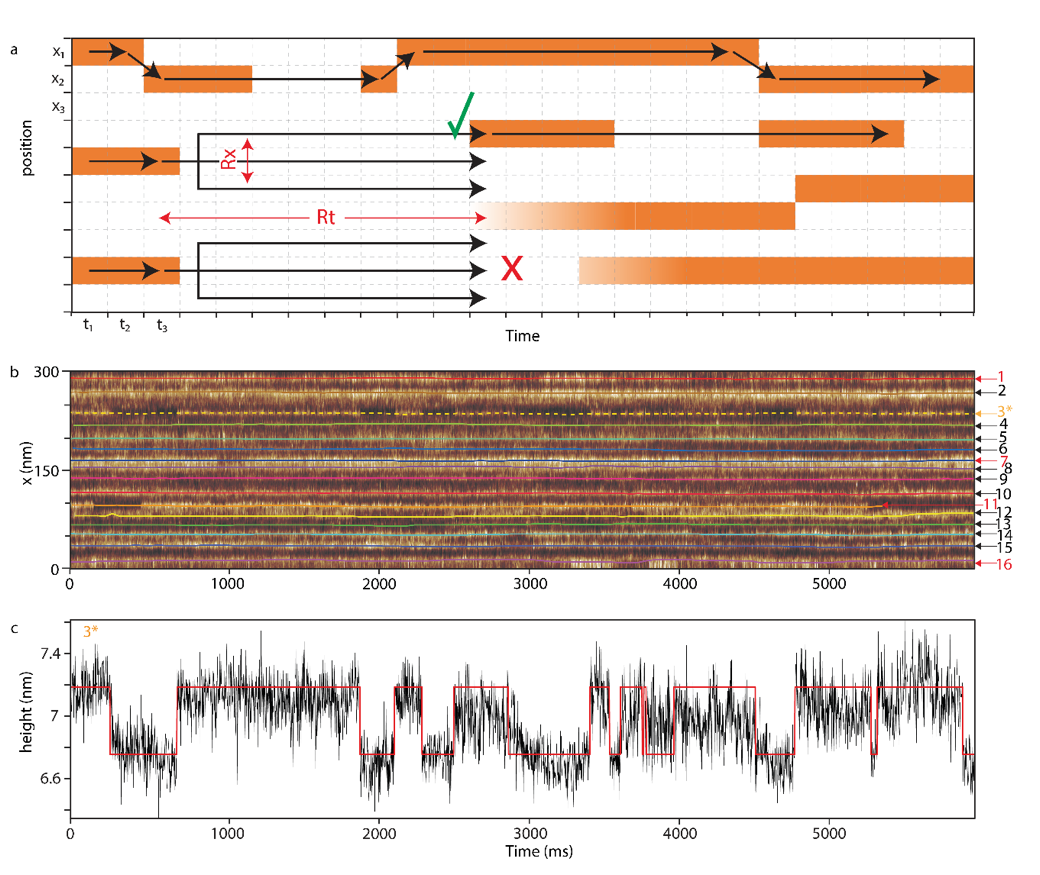** |
| --- |
| **Supplementary Figure S3)** **Protomer tracking and height/time trace signal analysis of HS-AFM-LS kymographs. a)** Schematic of a HS-AFM-LS kymograph showing various scenarios encountered by the protomer tracking algorithm. Protomer positions *x* and times *t* (orange filled) are linked using 1D-tracking with conditions on maximum drift *Rx*, and a maximum down time *Rt*. In this schematic *Rx* = +/-1 and *Rt* = 9, as depicted by the arrows (numbers chosen for this illustration). These conditions prevent incorrect tracking of different protomers during excursions. **b)** Example kymograph (6000ms) with overlaid tracking of 16 promoters (number labels are shown in black or red for active and inactive protomers, respectively. **c)** Extracted height/time trace of protomer #3 (dashed line in (b)) which is then analyzed by the state assignment algorithm (red line). |

**Supplementary Figure S4**

| **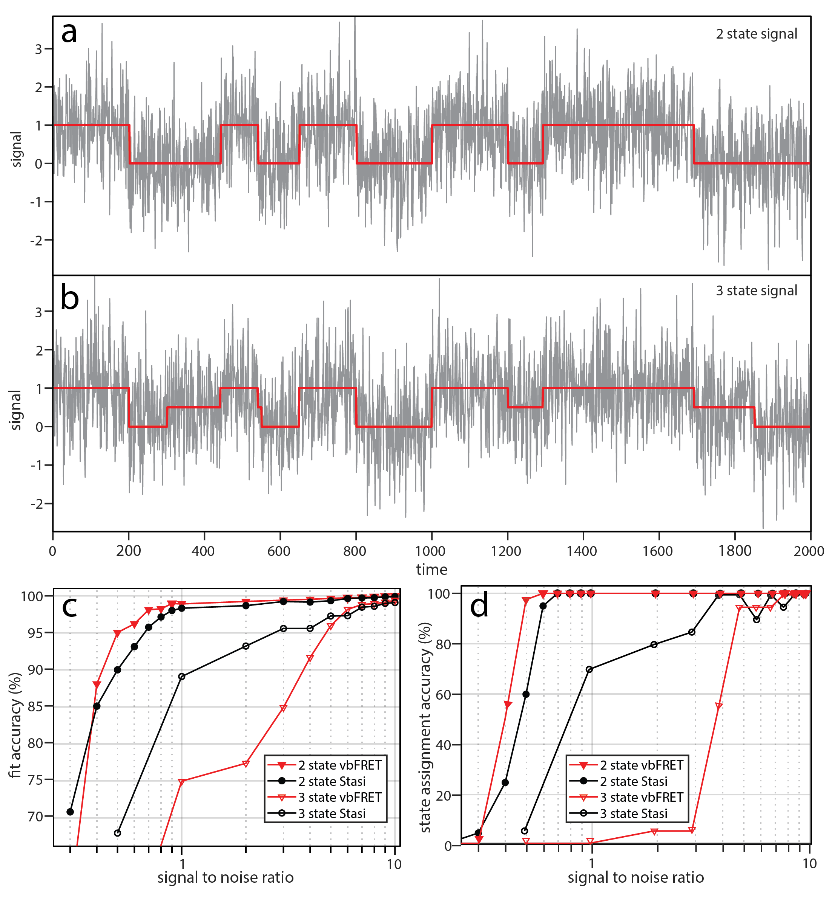** |
| --- |
| **Supplementary Figure S4) Performance of the Step Transition and State Identification (STaSI) algorithm for state-transition and state-number assignment - a comparison with vbFRET**. Simulated (**a)** two and (**b)** three state signals with added gaussian noise. The STaSI and vbFRET algorithms were tested by adding varying amounts of noise to the simulated signals to assess the (**c)** state-transition fit accuracy and (**d)** state-number assignment accuracy for two- (open markers) and three- (filled markers) state traces. Fit accuracy of STaSI (black) and vbFRET (red) was determined by considering each data point in the fit and comparing it to the original noise free signal. If the fitted data point was closest to the correct state in the original signal then this would be marked as accurate, otherwise the point is marked as inaccurate. For each fit, the fit accuracy is given as the % of data points in the fit that are correct. State assignment accuracy was defined as the % of time states were correctly allocated. Data points in c) and d) are averages of 20 tests at each signal-to-noise ratio. |

**Supplementary Figure S5**

| 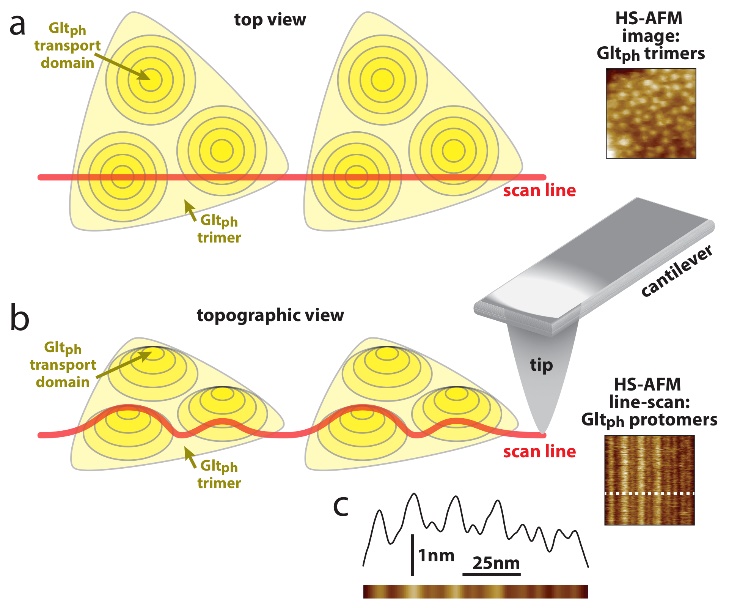 |
| --- |
| **Supplementary Figure S5) HS-AFM-LS contours Glt_Ph_ protomers at different height levels. a)** Schematic top view of Glt_Ph_ trimers (yellow) and scan line (red) which crosses some protomers on the highest point and others on the skirted area of each protomer. **b)** Topographical view of a) featuring schematics of a topographic HS-AFM-LS profile and the cantilever with tip. Inset images show an example HS-AFM image (top right) and HS-AFM-LS kymograph (bottom right) of protomers. **c)** Average height (bottom) and profile (top) displaying the varying heights of protomers when line-scanned by the tip. |

**Supplementary Figure S6**

| 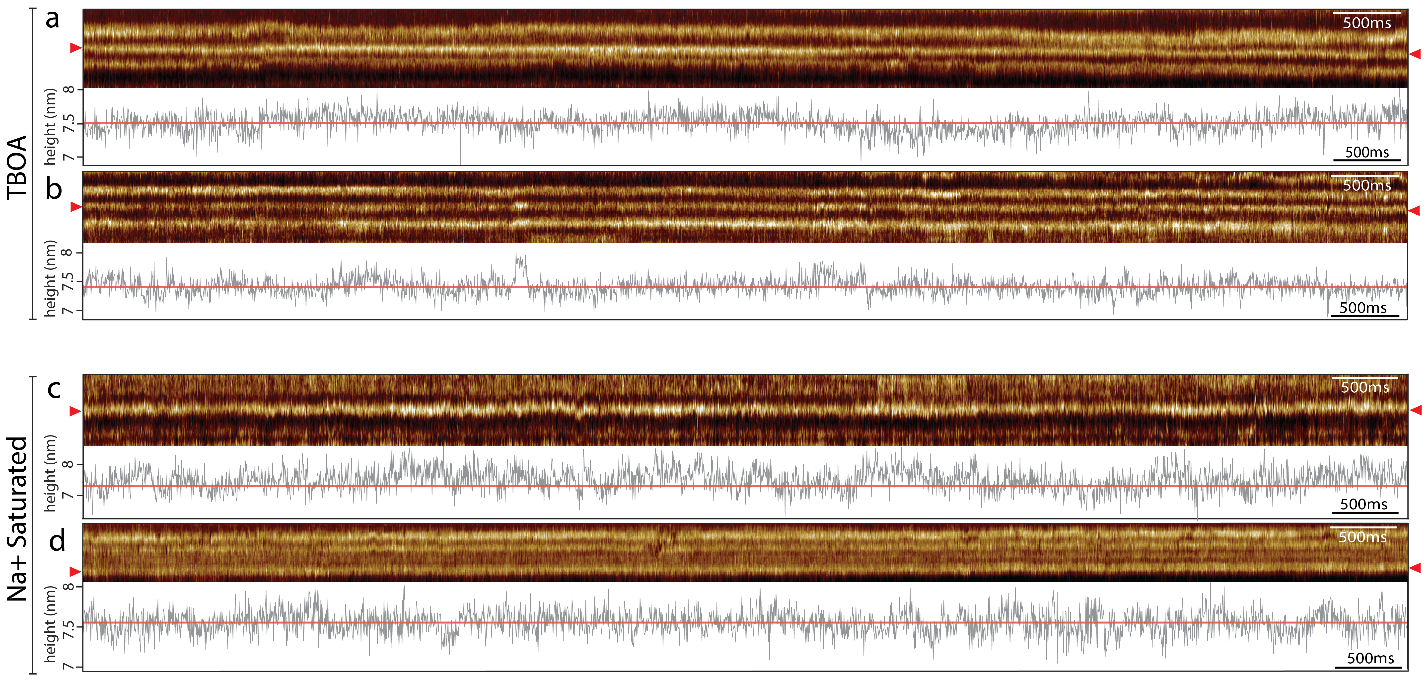 |
| --- |
| **Supplementary Figure S6) Glt_Ph_ transport domains stall in inhibitory conditions.** HS-AFM-LS raw data kymographs recorded in presence of the inhibitor TBOA (**a, b)** and in saturating Na^+^ (**c,d)** condition. All kymographs are recorded at 3.3ms temporal resolution. In all kymographs (a, b, c, d), one protomer signal has been isolated (grey) and fitted (red). In inhibitory conditions, the automated state detection algorithm suggest the existence of a single state only. |

**Supplementary Figure S7**

| **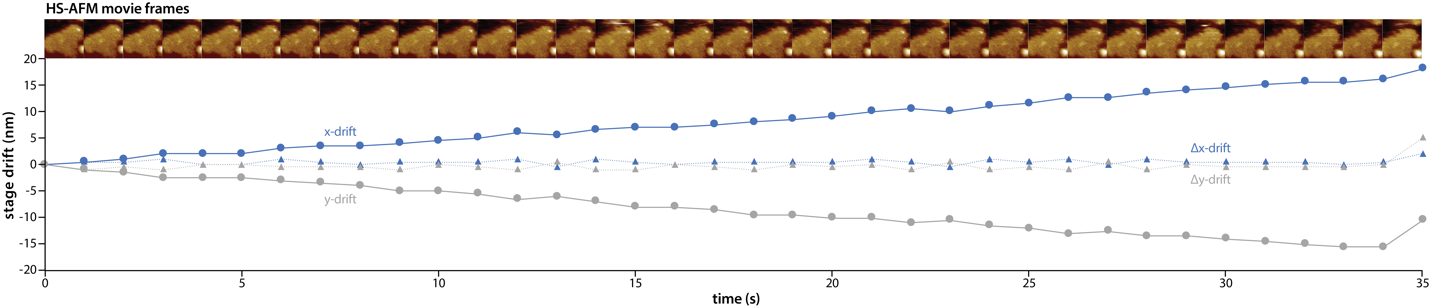** |
| --- |
| **Supplementary Figure S7) HS-AFM stage drift**. Top: HS-AFM image series over 35s of Glt_Ph_ membrane shown in figure 1c. Bottom: x,y stage drift analysis: In this particular experiment the stage drift was ~0.5nm/s. After extended experimental time, when the HS-AFM scanners and the entire HS-AFM setup are equilibrated, stage drift can be as little as 0.02nm/s (HeathREF). |

**Supplementary Figure S8**

| **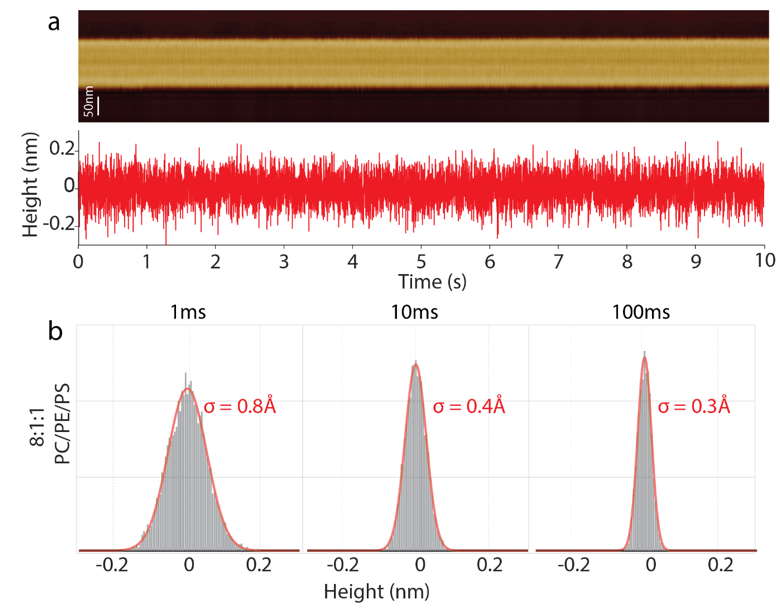** |
| --- |
| **Supplementary Figure S8) Noise analysis of HS-AFM-LS on membrane**. **a)** Top: HS-AFM-LS kymograph and, bottom: corresponding height/time trace. **b)** Height histograms with Gaussian fits and standard deviation (σ) values for the distribution of heights over 10 seconds, without filter (left), 10ms lowpass (middle) and 100ms lowpass (right). HS-AFM-LS measurements were performed on DOPC/DOPE/DOPS (8:1:1) membranes, the lipids in which Glt_Ph_ is reconstituted, at 1ms/line. |

**Supplementary Figure S9**

| **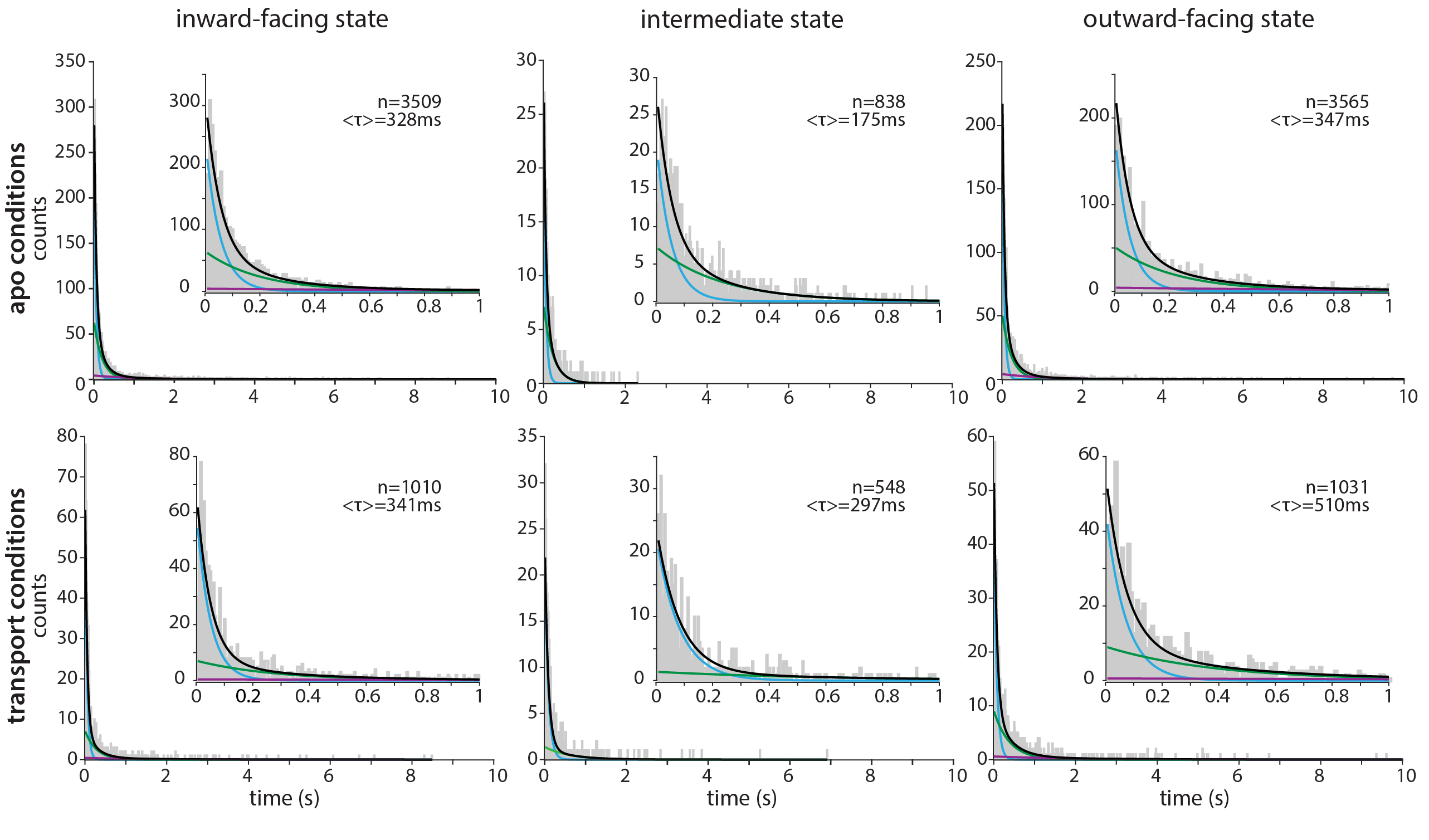** |
| --- |
| **Supplementary Figure S9) Dynamics of the Glt_Ph_ transport cycle (Linear Representation).** Distributions of detected dwell-times transport domain spent in each state in the absence (apo conditions, top) and presence of substrates (transport conditions, bottom): Inward- (left), intermediate (middle) and outward- (right) facing state dwell-times. All histograms (full length and insets) were linearly binned. The histograms were fitted (black) with multiple exponential components (blue, green and purple) and the corresponding exponential decay values and occurrence percentages are as indicated in Fig. 4a of the main text. The total number of transitions (*n*) and the average dwell-times (<τ>) are indicated in the top right of each panel. |

**Supplementary Table S1**

| **Protein** | **Organism** | **Function** |
| --- | --- | --- |
| AE1 | human | carbon-dioxide transporter(*1*) |
| ASCT2 | human | Neutral amino acid transporter(*2-4*) |
| EAAC1 | human | excitatory amino acid transporter(*5*) |
| EAAT1 | human | excitatory amino acid transporter(*6, 7*) |
| GLT-1 | human | Na^+^-Glu transporter(*8*) |
| hCNT1 | human | Nucleoside (pyrimidine and purine) transporter(*9*) |
| NaPi-II | human | Na^+^-phosphate transporter(*10, 11*) |
| SLC28 | human | Na^+^-dependent nucleoside transporters(*12*) |
| ASBT_NM_ | bacteria | bile acid sodium symporter(*13*) |
| ASBT_Yf_ | bacteria | bile acid sodium symporter(*14*) |
| BicA | bacteria | bicarbonate transporter(*15*) |
| bcChbC | bacteria | saccharide transporter(*16*) |
| CitS | bacteria | Na^+^-citrate symporter(*17*) |
| KpCitS | bacteria | Na^+^-dependent citrate transporter. |
| SeCitS | bacteria | citrate transporter(*18*) |
| CNT_NW_ | bacteria | nucleoside transporter(*19*) |
| _VC_CNT | bacteria | concentrative nucleoside transporter(*20, 21*) |
| ECF | bacteria | nickel/cobalt transporters (*22, 23*) |
| Glt_Ph_ | bacteria | Na^+^-Asp transporter(*24-27*) |
| Glt_Tk_ | bacteria | Na^+^-Asp transporter(*28-31*) |
| MalT | bacteria | Sugar transporter(*32*) |
| bcMalT | bacteria | Sugar transporter(*33*) |
| bcMalT-EIIC | bacteria | Sugar transporter(*34*) |
| bcMalT-EIIC-PTS | bacteria | Sugar transporter(*35*) |
| MtrF | bacteria | antimetabolite transporter(*36*) |
| NhaA | bacteria | Na^+^/H^+^ antiporter(*37-39*) |
| EcNhaA | bacteria | Na^+^/H^+^ antiporter (*40-42*) |
| MjNhaP1 | bacteria | Na^+^/H^+^ antiporter (*43*) |
| PaNhaP | bacteria | Na^+^/H^+^ antiporter (*44*) |
| NapA | bacteria | Na^+^/H^+^ exchanger(*39, 45*) |
| TtNapA | bacteria | Na^+^/H^+^ antiporter(*38, 45*) |
| TtCcdA | bacteria | electrons transporter(*46*) |
| SLC26Dg | bacteria | anion transporter(*47*) |
| ecUlaA | bacteria | vitamin-C transporter(*48*) |
| pmUlaA-EIIC | bacteria | Sugar transporter(*34*) |
| UraA | bacteria | uracil transporter(*49*) |
| VcINDY | bacteria | Na^+^-succinate transporter(*50*) |
| YdaH | bacteria | p-aminobenzoyl-glutamate transporter(*51*) |
| ECF | non bacteria | energy-coupling factor transporter(*52-58*) |
| Bor1 | plants | borate transporter(*33*) |
| UapA-G411VΔ1-11 | fungus | uric acid/xanthine H^+^-symporter(*59*) |

**Supplementary Table S1) List of transport proteins displaying the elevator mechanism.** List of proteins reported to use the elevator transporter mechanism.

**Supplementary Table S2**

|  | **outward** | | **intermediate** | | **inward** | | **active protomers** | **transition frequency**  **of active** | **protomer activity** | **total tracking time** |
| --- | --- | --- | --- | --- | --- | --- | --- | --- | --- | --- |
|  | **τ (*ms*)** | **P (*%*)** | **τ (*ms*)** | **P (*%*)** | **τ (*ms*)** | **P (*%*)** |  |  |  |  |
| **Apo** | 347 | 50%  (77%) | 175 | 5.2%  (2.5%) | 328 | 45%  (21%) | 55%  (n=340) | 1.4 s^-1^ | 0.77 s^-1^ | 100 min |
| **Transport** | 510 | 51%  (93%) | 297 | 11%  (1.9%) | 341 | 38%  (5.2%) | 23%  (n=422) | 1.05 s^-1^ | 0.24 s^-1^ | 153 min |
| **Na+ Saturated** | 1160 | 82%  (99%) | - | - | 330 | 18%  (1.2%) | 12%  (n=75) | 0.64 s^-1^ | 0.077 s^-1^ | 36 min |
| **TBOA** | 960 | 68%  (99%) | - | - | 701 | 32%  (0.6%) | 2.9%  (n=68) | 0.40 s^-1^ | 0.012 s^-1^ | 27 min |
| **Supplementary Table S2) Protomer activity, state occupancies and mean dwell-times. Left side columns**) Clear columns: average dwell-times (ms) of protomers in outward-, intermediate and inward-facing states. Shaded columns: probability of finding an active protomer in the particular state. Percentages in brackets indicate probability of finding any protomer (active or not during tracking time) in the particular state. **Right side columns)** Active protomers: fraction of protomers that exhibited elevator movement during observation in each condition. Transition frequency of active: frequency of activity of active protomers. Protomer activity: frequency of activity of all protomers. Total tracking time: collective detection time of protomers in each condition. | | | | | | | | | | |

**Supplementary Table S3**

|  | **path** | **out →** | **int** | **→ inw** | **inw →** | **int** | **→ out** | **out →** | **int** | **→ out** | **inw →** | **int** | **→ inw** |
| --- | --- | --- | --- | --- | --- | --- | --- | --- | --- | --- | --- | --- | --- |
| **apo** | **<τ> (*ms*)** | 210  (±49) | 179  (±17) | 227  (±56) | 209  (±27) | 168  (±17) | 182  (±30) | 345  (±33) | 183  (±16) | 354  (±38) | 349  (±36) | 161  (±13) | 336  (±34) |
|  | **P (*%*)** | 16.5%  (135) | | | 15.8%  (129) | | | 37.0%  (302) | | | 30.6%  (250) | | |
| **transport** | **<τ> (*ms*)** | 240  (±54) | 443  (±88) | 186  (±33) | 241  (±54) | 332  (±69) | 289  (±74) | 469  (±62) | 201  (±39) | 388  (±47) | 507  (±77) | 294  (±35) | 536  (±92) |
|  | **P (*%*)** | 15.8%  (84) | | | 15.2%  (81) | | | 37.0%  (197) | | | 32.0%  (170) | | |
| **graphical**  **summary** | 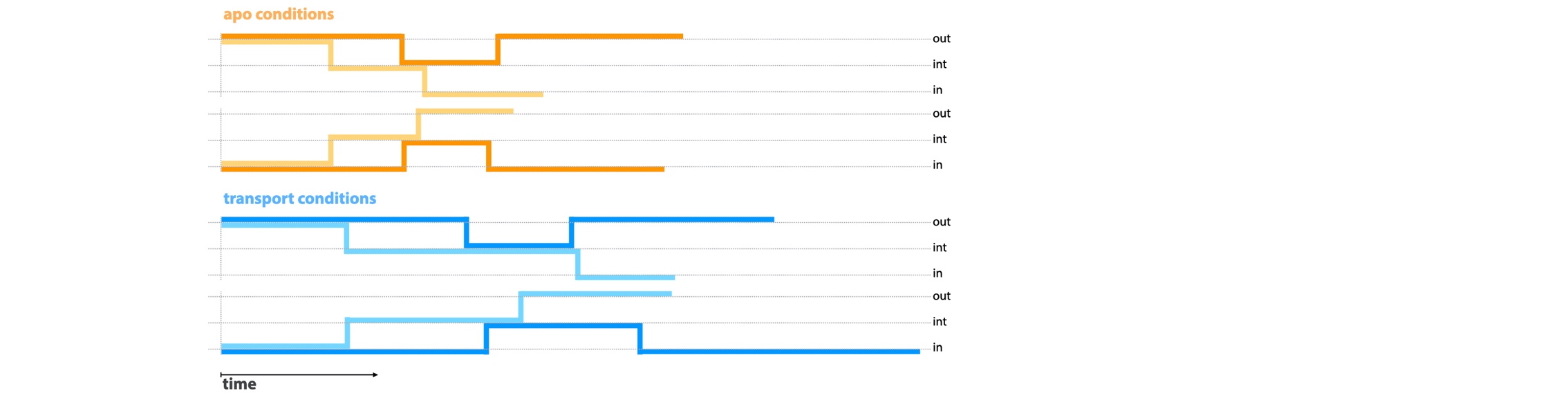 | | | | | | | | | | | | |
| **Supplementary Table S3) 2-step tracking of protomers visiting the intermediate state.** τ (ms): dwell-times in each state (top to bottom) for the sequence illustrated on top of the column. In brakets: Standard error (SE). P (%): probability of an active protomer taking a particular path. In brakets: Number of observations (n). Visiting the intermediate state as as failed attempt (returning to the initial position) is found ~2 times more often and is preceded by dwell-times ~2 times longer than in transit. Bottom: Graphical illustration of the data. | | | | | | | | | | | | | |

**Supplementary Movies Captions**

| 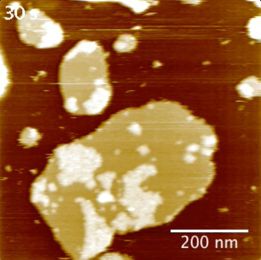 | **Supplementary Movie 1)**  **Surface adsorption and morphology of Glt_Ph_ reconstituted vesicles.**  HS-AFM movie of Glt_Ph_ reconstituted densely in DOPC:DOPE:DOPS (8:1:1 w:w:w) membranes, imaged in substrate free (apo) conditions.  **Movie parameters:** frame size: 300 pixels, image size: 670 nm, full false color scale: 46nm, scan speed: 1 frame/s | |
| --- | --- | --- |
| 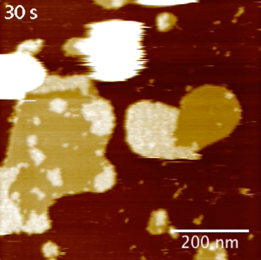 | **Supplementary Movie 2)**  **Surface adsorption and morphology of Glt_Ph_ reconstituted vesicles.**  HS-AFM movie of Glt_Ph_ reconstituted densely in DOPC:DOPE:DOPS (8:1:1 w:w:w) membranes, imaged in substrate free (apo) conditions.  **Movie parameters:** frame size: 300 pixels, image size: 670nm, full false color scale: 46nm, scan speed: 1 frame/s | |
| 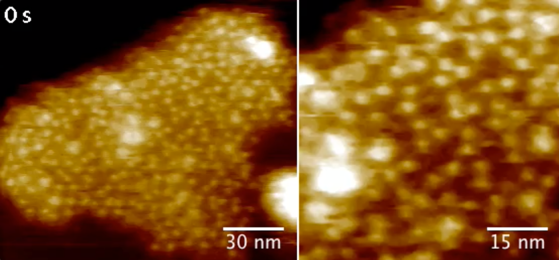 | | **Supplementary Movie 3)**  **Glt_Ph_ packing and activity in membranes.**  HS-AFM movie of HS-AFM movie of Glt_Ph_ reconstituted densely in a DOPC:DOPE:DOPS (8:1:1 w:w:w) membranes, imaged in substrate free (apo) conditions.  **Movie parameters:** frame size: 300 pixels, image size: 150, full false color scale: 10nm, scan speed: 1 frame/s |
| 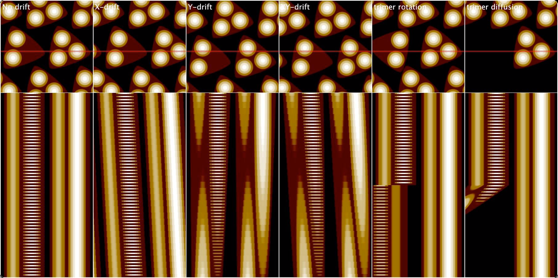 | | **Supplementary Movie 4) Animation on possible drifts in the system and the outcome in line scanning kymographs.**  Top panels demonstrating various drift scenarios while 2D scanning. On the bottom, the progression of kymographs constructed while line-scanning corresponding the drifts showed on the top. |

**Supplementary MATLAB Codes**

S0_LS_Image_analysis.m

%% Code to analyze kymograph images to output height vs time data for each high feature

%left to right should equal the time domain

%Step 1

%run code - check output image that features have been tracked correctly,

%if not then alter below setting to optimize tracking.

%For lateral drift compensation use S1_1_Kymo_align after running this code

%then re-run this code to analyze the drift corrected kymograph.

%If tracks are needed to be removed use S1_2_track_remover or

%Step 2

%Go to S1_LS_Track_Analyzer

avx = 800; %pixel average in time domain

max_g = 2000; %maximum down time (pix)

max_drift = 2; %maxium +/- pixels to search for in x

edg = 5; %exclude protomers with edg pixels

min_length = 1000; %minimum protomers track length (pixels)

thresh = 0.8; %ignore traces with heights below thresh % of maximum

%%

if exist('kymo')==0

prompt = 'Open new image (0 for no, 1 for yes)? ';

new = input(prompt);

if new ==1

[f,path] = uigetfile('*.tif');

f = fullfile(path,f); %tif (in nm) filename

end

end

if exist('kymo')==0

clearvars -except max_g avx min_length edg max_drift f thresh kymo time t_n

A = imread(f);

t_n = numel(A(1,:));

x_n = numel(A(:,1));

sz = size(A);

time =linspace(1,t_n,t_n)';

else

clearvars -except A As avx avy f time t_n max_g min_length edg max_drift thresh kymo

A = As;

x_n = numel(A(:,1));

sz = size(A);

time =linspace(1,t_n,t_n)';

end

Amx = zeros(sz);

for i = 1:x_n

Amx(i,:) = movmean(A(i,:),avx)-min(Amx(:));

end

Amx = Amx.*(Amx>thresh*max(Amx(:)));

Aall = reshape(Amx, [1, x_n*t_n]); %combines data into single colunm

[h,locs,wd] = findpeaks(Aall,1,'MinPeakProminence',0.01,...

'MinPeakDistance',2,...

'WidthReference','halfheight');

%'Annotate','extents');

peaks_n = numel(wd);

temptime = zeros(1,peaks_n);

%reassemble

for i = 1:t_n

for j = 1:peaks_n

if (locs(j) > (i-1)*x_n) && locs(j) < i*x_n

temptime(:,j) = i;

end

end

end

locs = locs - x_n*(temptime-1);

for i = 1:t_n

pos = temptime>(i-0.1) & temptime<(i+1);

struc = locs(pos)';

struc_wd = wd(pos)';

CC{i} = struc;

CC_wd{i} = struc_wd;

end

CC = CC';

CC_wd = CC_wd';

%Track peaks

tracks = simpletracker(CC,'Method','hungarian','MaxLinkingDistance',max_drift,'MaxGapClosing',max_g);

n_tracks = numel(tracks)

tracked = zeros(t_n,n_tracks);

trackedN = tracked; tracked_wd = tracked; trackedN_wd = tracked;

for i_track = 1:n_tracks

pos = (tracks{i_track,1});

for j = 1:t_n

if pos(j)>0

tracked(j,i_track) = CC{j,1}(pos(j)); trackedN(j,i_track) = CC{j,1}(pos(j));

tracked_wd(j,i_track) = CC_wd{j,1}(pos(j)); trackedN_wd(j,i_track) = CC_wd{j,1}(pos(j));

else

tracked(j,i_track) = 0;

trackedN(j,i_track) = NaN;

tracked_wd(j,i_track) = 0;

trackedN_wd(j,i_track) = NaN;

end

end

end

y = tracked;

for j = 1:n_tracks %creat tracks with start-finish points and

for i = 1:t_n

if sum(y(1:i,j))> 0.9 && sum(y(i:numel(y(:,1)),j))> 0.9

yt(i,j) = 1;

else

yt(i,j) = 0;

end

end

end

for j = 1:n_tracks

t1 = time(yt(:,j)>0.5);

track_time{j} = t1;

track_x{j} = trackedN(t1,j);

track_x{j} = fillmissing(track_x{j},'movmean',max_g,'EndValues','nearest');

track_x{j} = round(movmean(track_x{j},100));

track_wd{j} = trackedN_wd(t1,j);

track_wd{j} = fillmissing(track_wd{j},'nearest');

track_wd{j} = movmean(track_wd{j},100);

track_xu{j} = track_x{j}+ round(track_wd{j}/5);

track_xd{j} = track_x{j}- round(track_wd{j}/5);

end

for j = 1:n_tracks

for t_i = 1:numel(track_time{j})

if track_x{j}< x_n- edg & track_x{j} > edg & numel(track_x{j}) >10 & sum(tracked(:,j) > 0.5) > min_length %exclude boundary tracks and tracks shorter than 300 pixels

track_hi{j}(t_i) = A(track_x{j}(t_i),track_time{j}(t_i));

track_hwd{j}(t_i) = mean(A(track_xd{j}(t_i):track_xu{j}(t_i),track_time{j}(t_i)))';

else

track_x{j} = [];

track_time{j} = [];

track_wd{j} = [];

end

end

end

clearvars CC CC_wd h locs pos temptime yt y wd trackedN trackedN_wd tracked_wd time Aall t1 ti i i_track j peaks_n struc struc_wd

figure(2)

imagesc(A)

load('lutafm.mat')

colormap(lutafm)

hold on

for j = 1:n_tracks

%if track_x{j}>0

text(min(track_time{1,j})+50,min(track_x{1,j}),num2str(j),'Color','c')

plot(track_time{1,j},track_x{1,j},'LineWidth',1)

%end

end

-----------------------------------------------------------------------------

S1__Kymo_align.m

%%Code to align kymograph base on the tracking of one protomer found using

%%S0_LS_Image analysis

% After running this code re-run S0_LS_Image analysis

track = 2; %align based on track

%%

kymo = 1;

mx_shift = max(track_x{track})-min(track_x{track});

As = zeros(sz(1)+mx_shift+1,sz(2));

shift = max(track_x{track})-track_x{track} +1;

for i = 1:t_n

As(shift(i):shift(i)+sz(1)-1,i) = A(1:sz(1),i);

end

As = As-min(As(:));

-----------------------------------------------------------------------------

S1_2_track_remover.m

polytracks = [6];

fitpoly = zeros(n_tracks,1);

fitpoly(polytracks) = 1;

for j = 1:n_tracks

if fitpoly(j,1)>0

track_hwd{j} = [];

track_x{j} = [];

track_time{j} = [];

else

end

end

-----------------------------------------------------------------------------

S1_3_track_fill.m

polytracks = [3];

fitpoly = zeros(n_tracks,1);

fitpoly(polytracks) = 1;

for j = 1:n_tracks

if fitpoly(j,1)>0

for i = 1:t_n

track_time{j}(i) = i;

track_x{j}(i) = round(mean(track_x{j}));

track_xd{j}(i) = round(mean(track_x{j})-2);

track_xu{j}(i) = round(mean(track_x{j})+2);

end

else

end

end

for j = 1:n_tracks

if fitpoly(j,1)>0

for i = 1:t_n

track_hwd{j}(i) = mean(A(track_xd{j}(i):track_xu{j}(i),track_time{j}(i)))';

end

else

end

end

-----------------------------------------------------------------------------

S2_LS_Track_Analyzer.m

%%Code to perform state analysis of height vs time traces extracted from kymograph

%input polynomial order into poly_all to apply a polynomial fitting to z

%data to all tracks. Use poly and polytracks to input polynomial to apply

%polynomial fitting to a single track.

%Use 'Quality' setting to exclude tracks with low signal to noise ratio

%dwell times for all traces output as 'all_down_wd', 'all_mid_wd' and 'all_up_wd'

%dwell heights for all traces output as 'all_down_h', 'all_mid_h' and 'all_up_h'

%normalized dwell heights for all traces output as 'all_down_h_n', 'all_mid_h_n' and 'all_up_h_n'

%Use S2_1_Plot_Track to plot single traces

Quality = 1.35;

stasi = 1; %perform stasi?

poly_all = 0;

poly = 0; %selective poly

polytracks = []; %select track

%%

fitpoly = zeros(n_tracks,1);

fitpoly(polytracks) = 1;

if stasi == 1

for j = 1:n_tracks

if track_x{j}< x_n-edg & sum(track_x{j} >edg)>0 & numel(track_x{j}) >5 & sum(tracked(:,j) > 0.5) > 300

for i = 1:numel(track_hwd{j})-1

delt_h{j}(i) = track_hwd{j}(i)-track_hwd{j}(i+1);

end

track_sd{j} = std(delt_h{j});

[p,sii,mu] = polyfit((1:numel(track_hwd{j})'),track_hwd{j},poly_all); %polynomial fit

f_z = polyval(p,(1:numel(track_hwd{j}))',[],mu);

track_hwd_p{j} = (track_hwd{j}' -f_z+mean(f_z))';

output(j,:) = StaSI_inputeff(track_hwd_p{j}, track_sd{j});%polynomial fit subtractmin_h(j) = min(track_hwd{j});

%output(j,:) = StaSI_inputeff(track_hwd{j}, track_sd{j});

max_h(j,1) = max(track_hwd_p{j});

var_h(j,1) = var(track_hwd_p{j});

end

end

err_mult =1.4;

for j = 1:n_tracks

if fitpoly(j,1)>0

[p,sii,mu] = polyfit((1:numel(track_hwd{j})'),track_hwd{j},poly); %polynomial fit

f_z = polyval(p,(1:numel(track_hwd{j}))',[],mu);

track_hwd_p{j} = (track_hwd{j}' -f_z +mean(f_z))';

output(j,:) = StaSI_inputeff(track_hwd_p{j}, track_sd{j});%polynomial fit subtractmin_h(j) = min(track_hwd{j});

%output(j,:) = StaSI_inputeff(track_hwd{j}, track_sd{j});

max_h(j,1) = max(track_hwd_p{j});

var_h(j,1) = var(track_hwd_p{j});

else

end

end

for i = 1:numel(output)

if output(i).breaks >0.5

[minMDL(i,2),minMDL(i,1)] = min(output(i).MDL);

end

end

end

s2 = [];

s3 = [];

for j = 1:numel(output)

if output(j).breaks >0.5 & numel(track_x{j}) & numel(track_hwd_p{j})

%2state

stasi_2{j} = output(j).eff_fit(2,:);

[s2{j}.up_h, s2{j}.up_wd, s2{j}.down_h, s2{j}.down_wd, s2{j}.ideal, s2{j}.ideal_i]=TwoState_measure(track_time{j}, track_hwd_p{j},stasi_2{j});

[s2{j}.up_h, s2{j}.up_wd, s2{j}.down_h, s2{j}.down_wd, s2{j}.ideal, s2{j}.ideal_i]=TwoState_measure(track_time{j}, track_hwd_p{j},s2{j}.ideal);

[s2{j}.up_h, s2{j}.up_wd, s2{j}.down_h, s2{j}.down_wd, s2{j}.ideal, s2{j}.ideal_i]=TwoState_measure(track_time{j}, track_hwd_p{j},s2{j}.ideal);

%3state

stasi_3{j} = output(j).eff_fit(3,:);

[s3{j}.up_h, s3{j}.up_wd, s3{j}.mid_h, s3{j}.mid_wd, s3{j}.down_h, s3{j}.down_wd, s3{j}.ideal, s3{j}.ideal_i]=ThreeState_measure(track_time{j}, track_hwd_p{j},stasi_3{j});

[s3{j}.up_h, s3{j}.up_wd, s3{j}.mid_h, s3{j}.mid_wd, s3{j}.down_h, s3{j}.down_wd, s3{j}.ideal, s3{j}.ideal_i]=ThreeState_measure(track_time{j}, track_hwd_p{j},s3{j}.ideal);

[s3{j}.up_h, s3{j}.up_wd, s3{j}.mid_h, s3{j}.mid_wd, s3{j}.down_h, s3{j}.down_wd, s3{j}.ideal, s3{j}.ideal_i]=ThreeState_measure(track_time{j}, track_hwd_p{j},s3{j}.ideal);

[states(j,1), states(j,2)] = State_numbers(track_hwd_p{j}, s2{j}.ideal, s3{j}.ideal,err_mult);

states(j,3) = (max(output(j).eff_fit(2,:))) - (min(output(j).eff_fit(2,:)));

states(j,4) = var(track_hwd_p{j});

[states(j,6) ,states(j,5)] = min(output(j).MDL);

states(j,7) = j;

states(j,8) = track_sd{j};

states(j,9) = states(j,3)/track_sd{j};

end

end

s2 = [];

s3 = [];

for j = 1:numel(output)

if states(j,2)>0 && output(j).breaks >0.5

%2state

stasi_2{j} = output(j).eff_fit(2,:);

stasi_3{j} = output(j).eff_fit(3,:);

states(j,3) = (max(output(j).eff_fit(2,:))) - (min(output(j).eff_fit(2,:)));

for i = 1:numel(track_hwd{j})-1

delt_h{j}(i) = track_hwd{j}(i)-track_hwd{j}(i+1);

end

track_sd{j} = std(delt_h{j});

states(j,8) = track_sd{j};

states(j,9) = states(j,3)/track_sd{j};

stasi_2_shift{j} = ThreeState_ToTwo_v2(track_time{j}, track_hwd{j},stasi_3{j});

[s2{j}.up_h, s2{j}.up_wd, s2{j}.down_h, s2{j}.down_wd, s2{j}.ideal, s2{j}.ideal_i]=TwoState_measure(track_time{j}, track_hwd_p{j},stasi_2_shift{j});

[s2{j}.up_h, s2{j}.up_wd, s2{j}.down_h, s2{j}.down_wd, s2{j}.ideal, s2{j}.ideal_i]=TwoState_measure(track_time{j}, track_hwd_p{j},s2{j}.ideal);

s2{j}.up_h =[]; s2{j}.up_wd =[];s2{j}.down_h =[]; s2{j}.down_wd =[];

[s2{j}.up_h, s2{j}.up_wd, s2{j}.down_h, s2{j}.down_wd, s2{j}.ideal, s2{j}.ideal_i]=TwoState_measure(track_time{j}, track_hwd_p{j},s2{j}.ideal);

states(j,10) = mean((track_hwd_p{j}-s2{j}.ideal).^2).^0.5;

%3state

[s3{j}.up_h, s3{j}.up_wd, s3{j}.mid_h, s3{j}.mid_wd, s3{j}.down_h, s3{j}.down_wd, s3{j}.ideal, s3{j}.ideal_i]=ThreeState_measure(track_time{j}, track_hwd_p{j},stasi_3{j});

[s3{j}.up_h, s3{j}.up_wd, s3{j}.mid_h, s3{j}.mid_wd, s3{j}.down_h, s3{j}.down_wd, s3{j}.ideal, s3{j}.ideal_i]=ThreeState_measure(track_time{j}, track_hwd_p{j},s3{j}.ideal);

[s3{j}.up_h, s3{j}.up_wd, s3{j}.mid_h, s3{j}.mid_wd, s3{j}.down_h, s3{j}.down_wd, s3{j}.ideal, s3{j}.ideal_i]=ThreeState_measure(track_time{j}, track_hwd_p{j},s3{j}.ideal);

end

end

all_up_h = [];all_down_h =[];all_up_wd = [];all_down_wd =[];

all_mid_h = []; all_mid_wd = []; all_up_h_n = []; all_down_h_n =[]; all_mid_h_n = [];

for i = 1:numel(states(:,1))

%% 2 state analyis

if states(i,9) > Quality && states(i,2) ==2

all_up_h = vertcat(all_up_h,s2{i}.up_h(:));

all_up_h_n = vertcat(all_up_h_n,(s2{i}.up_h(:)-mean(s2{i}.down_h(:)))/states(i,3));

all_up_wd = vertcat(all_up_wd,s2{i}.up_wd(:));

all_down_h = vertcat(all_down_h,s2{i}.down_h(:));

all_down_h_n = vertcat(all_down_h_n,(s2{i}.down_h(:)-mean(s2{i}.down_h(:)))/states(i,3));

all_down_wd = vertcat(all_down_wd,s2{i}.down_wd(:));

end

%% 3 state analysis

if states(i,9) > Quality && states(i,2) ==3

all_up_h = vertcat(all_up_h,s3{i}.up_h(:));

all_up_h_n = vertcat(all_up_h_n,(s3{i}.up_h(:)-mean(s3{i}.down_h(:)))/states(i,3));

all_up_wd = vertcat(all_up_wd,s3{i}.up_wd(:));

all_mid_h = vertcat(all_mid_h,s3{i}.mid_h(:));

all_mid_h_n = vertcat(all_mid_h_n,(s3{i}.mid_h(:)-mean(s3{i}.down_h(:)))/states(i,3));

all_mid_wd = vertcat(all_mid_wd,s3{i}.mid_wd(:));

all_down_h = vertcat(all_down_h,s3{i}.down_h(:));

all_down_h_n = vertcat(all_down_h_n,(s3{i}.down_h(:)-mean(s3{i}.down_h(:)))/states(i,3));

all_down_wd = vertcat(all_down_wd,s3{i}.down_wd(:));

end

end

%%

non_act = sum(states(:,2) == 1);

act = sum(states(:,2) > 1);

load('lutafm.mat')

figure(7)

imagesc(A)

colormap(lutafm)

hold on

for j = 1:numel(output)

if states(j,2) >1

plot(track_time{1,j},track_x{1,j},'c','LineWidth',1)

%plot(track_time{1,j},track_xu{1,j},'r','LineWidth',0.5)

%plot(track_time{1,j},track_xd{1,j},'r','LineWidth',0.5)

%imagesc(min(track_time{1,j}),mean(track_x{1,j}),10*(s2{j}.ideal>min(s2{j}.ideal)))

text(min(track_time{1,j})+30,min(track_x{1,j}),num2str(j),'Color','c')

else

text(min(track_time{1,j})+30,min(track_x{1,j}),num2str(j),'Color','r')

plot(track_time{1,j},track_x{1,j},'r')

end

end

set(gca,'TickDir','out')

if act>0

figure(3)

ha = tight_subplot(2*act,1,[.0 .03],[.1 .01],[.1 .01]);

count = 0;

for track = 1:numel(output)

if states(track,2) >1 & track_x{track}< x_n-edg & sum(track_x{track} >edg)>0

count = count +1;

axes(ha(count));

imagesc(A((track_x{track}-10):(track_x{track}+10),track_time{track}),[min(track_hwd_p{track}) max(track_hwd_p{track})]);

colormap(lutafm)

set(gca,'TickDir','out')

ylabel(num2str(track))

hold on

imagesc(0,20,1*(s2{track}.ideal>min(s2{track}.ideal)))

set(gca,'TickDir','out')

count = count +1;

axes(ha(count));

imagesc(1*(s2{track}.ideal>min(s2{track}.ideal)))

set(gca,'TickDir','out')

hold on

plot(track_hwd_p{track},'r','LineWidth',1)

set(gca,'TickDir','out')

if states(track,2) ==2

plot(s2{track}.ideal,'b','LineWidth',1)

else if states(track,2)==3

plot(s3{track}.ideal,'g','LineWidth',2)

end

end

xlim([0 numel(track_time{track})])

ylim([min(track_hwd_p{track}) max(track_hwd_p{track})])

set(ha(1),'XTickLabel','')

set(gca,'Ydir','Normal')

set(gca,'TickDir','out')

end

end

set(ha(1:(act-1)),'XTickLabel','')

title('Active')

end

if non_act>0

figure(4)

ha = tight_subplot(2*non_act,1,[.0 .03],[.1 .01],[.1 .01]);

count = 0;

for track = 1:numel(output)

if states(track,2) < 2 & track_x{track}< x_n-edg & sum(track_x{track} >edg)>0

count = count +1;

axes(ha(count));

imagesc(A((track_x{track}-10):(track_x{track}+10),track_time{track}),[min(track_hwd_p{track}) max(track_hwd_p{track})]);

set(gca,'TickDir','out')

colormap(lutafm)

ylabel(num2str(track))

hold on

imagesc(0,20,1*(s2{track}.ideal>min(s2{track}.ideal)))

count = count +1;

axes(ha(count));

imagesc(1*(s2{track}.ideal>min(s2{track}.ideal)))

hold on

plot(track_hwd_p{track})

plot(output(track).eff_fit(1,:))

set(gca,'TickDir','out')

xlim([0 numel(track_time{track})])

ylim([min(track_hwd_p{track}) max(track_hwd_p{track})])

set(ha(1),'XTickLabel','')

set(gca,'Ydir','Normal')

end

end

set(ha(1:(non_act-1)),'XTickLabel','')

end

figure(5)

plot(all_down_wd,all_down_h_n,'o')

hold on

plot(all_up_wd,all_up_h_n,'o')

plot(all_mid_wd,all_mid_h_n,'o')

set(gca,'XScale','Log','TickDir','out')

xlabel('Dwell time')

ylabel('Normalized dwell height')

-----------------------------------------------------------------------------

S3_1_Plot_1_Track.m

track =2

figure(1)

plot(track_time{track}*0.0033,track_hwd_p{track}+7,'k')

hold on

plot(track_time{track}*0.0033,s2{track}.ideal+7,'r','LineWidth',2)

%plot(delt_h{track},'r')

%plot(track_time{track}*0.0033,output(track).eff_fit(1,:)+7,'r','LineWidth',2)

plot(track_time{1,track}*0.0033,s3{track}.ideal+7,'g','LineWidth',2)

%xlim([min(track_time{1,track})*0.0033 max(track_time{1,track})*0.0033])

xlabel('Time (s)')

ylabel('Height (nm)')

set(gca,'TickDir','out','fontsize', 16,'FontName', 'Arial','box','on')

**References**

1. E. Ficici, J. D. Faraldo-Gomez, M. L. Jennings, L. R. Forrest, Asymmetry of inverted-topology repeats in the AE1 anion exchanger suggests an elevator-like mechanism. *The Journal of general physiology* **149**, 1149-1164 (2017).

2. X. Yu *et al.*, Cryo-EM structures of the human glutamine transporter SLC1A5 (ASCT2) in the outward-facing conformation. *eLife* **8**, (2019).

3. A. A. Garaeva, A. Guskov, D. J. Slotboom, C. Paulino, A one-gate elevator mechanism for the human neutral amino acid transporter ASCT2. *Nature communications* **10**, 3427 (2019).

4. A. A. Garaeva *et al.*, Cryo-EM structure of the human neutral amino acid transporter ASCT2. *Nature structural & molecular biology* **25**, 515-521 (2018).

5. N. Rosental, A. Gameiro, C. Grewer, B. I. Kanner, A conserved aspartate residue located at the extracellular end of the binding pocket controls cation interactions in brain glutamate transporters. *The Journal of biological chemistry* **286**, 41381-41390 (2011).

6. R. J. Cater, R. J. Vandenberg, R. M. Ryan, Tuning the ion selectivity of glutamate transporter-associated uncoupled conductances. *The Journal of general physiology* **148**, 13-24 (2016).

7. M. H. Cheng, D. Torres-Salazar, A. D. Gonzalez-Suarez, S. G. Amara, I. Bahar, Substrate transport and anion permeation proceed through distinct pathways in glutamate transporters. *eLife* **6**, (2017).

8. N. Silverstein, T. J. Crisman, L. R. Forrest, B. I. Kanner, Cysteine scanning mutagenesis of transmembrane helix 3 of a brain glutamate transporter reveals two conformationally sensitive positions. *The Journal of biological chemistry* **288**, 964-973 (2013).

9. R. Mulinta, S. Y. M. Yao, A. M. L. Ng, C. E. Cass, J. D. Young, Substituted cysteine accessibility method (SCAM) analysis of the transport domain of human concentrative nucleoside transporter 3 (hCNT3) and other family members reveals features of structural and functional importance. *The Journal of biological chemistry* **292**, 9505-9522 (2017).

10. C. Fenollar-Ferrer, L. R. Forrest, Structural models of the NaPi-II sodium-phosphate cotransporters. *Pflugers Archiv : European journal of physiology* **471**, 43-52 (2019).

11. B. Gasnier, Watching the Pulleys Turn while the Elevator Moves in a Secondary Transporter. *Biophysical journal* **111**, 895-897 (2016).

12. S. Y. M. Yao, J. D. Young, Inward- and outward-facing homology modeling of human concentrative nucleoside transporter 3 (hCNT3) predicts an elevator-type transport mechanism. *Channels (Austin, Tex.)* **12**, 291-298 (2018).

13. N. J. Hu, S. Iwata, A. D. Cameron, D. Drew, Crystal structure of a bacterial homologue of the bile acid sodium symporter ASBT. *Nature* **478**, 408-411 (2011).

14. X. Zhou *et al.*, Structural basis of the alternating-access mechanism in a bile acid transporter. *Nature* **505**, 569-573 (2014).

15. C. Wang *et al.*, Structural mechanism of the active bicarbonate transporter from cyanobacteria. *Nature plants* **5**, 1184-1193 (2019).

16. Y. Cao *et al.*, Crystal structure of a phosphorylation-coupled saccharide transporter. *Nature* **473**, 50-54 (2011).

17. J. S. Lolkema, D. J. Slotboom, Structure and elevator mechanism of the Na(+)-citrate transporter CitS. *Current opinion in structural biology* **45**, 1-9 (2017).

18. D. Wöhlert, M. J. Grötzinger, W. Kühlbrandt, Ö. Yildiz, Mechanism of Na(+)-dependent citrate transport from the structure of an asymmetrical CitS dimer. *eLife* **4**, e09375 (2015).

19. M. Hirschi, Z. L. Johnson, S. Y. Lee, Visualizing multistep elevator-like transitions of a nucleoside transporter. *Nature* **545**, 66-70 (2017).

20. Z. L. Johnson, C. G. Cheong, S. Y. Lee, Crystal structure of a concentrative nucleoside transporter from Vibrio cholerae at 2.4 Å. *Nature* **483**, 489-493 (2012).

21. Z. L. Johnson *et al.*, Structural basis of nucleoside and nucleoside drug selectivity by concentrative nucleoside transporters. *eLife* **3**, e03604 (2014).

22. Y. Yu *et al.*, Planar substrate-binding site dictates the specificity of ECF-type nickel/cobalt transporters. *Cell research* **24**, 267-277 (2014).

23. Z. Bao *et al.*, Structure and mechanism of a group-I cobalt energy coupling factor transporter. *Cell research* **27**, 675-687 (2017).

24. Y. Ruan *et al.*, Direct visualization of glutamate transporter elevator mechanism by high-speed AFM. *Proceedings of the National Academy of Sciences of the United States of America* **114**, 1584-1588 (2017).

25. N. Akyuz *et al.*, Transport domain unlocking sets the uptake rate of an aspartate transporter. *Nature* **518**, 68 (2015).

26. J. Wang, T. Albers, C. Grewer, Energy Landscape of the Substrate Translocation Equilibrium of Plasma-Membrane Glutamate Transporters. *The journal of physical chemistry. B* **122**, 28-39 (2018).

27. A. Vergara-Jaque, C. Fenollar-Ferrer, D. Kaufmann, L. R. Forrest, Repeat-swap homology modeling of secondary active transporters: updated protocol and prediction of elevator-type mechanisms. *Frontiers in pharmacology* **6**, 183 (2015).

28. V. Arkhipova, A. Guskov, D. J. Slotboom, Structural ensemble of a glutamate transporter homologue in lipid nanodisc environment. *Nature communications* **11**, 998 (2020).

29. S. Jensen, A. Guskov, S. Rempel, I. Hänelt, D. J. Slotboom, Crystal structure of a substrate-free aspartate transporter. *Nature structural & molecular biology* **20**, 1224-1226 (2013).

30. A. Guskov, S. Jensen, I. Faustino, S. J. Marrink, D. J. Slotboom, Coupled binding mechanism of three sodium ions and aspartate in the glutamate transporter homologue Glt(Tk). *Nature communications* **7**, 13420 (2016).

31. V. Arkhipova *et al.*, Binding and transport of D-aspartate by the glutamate transporter homolog Glt(Tk). *eLife* **8**, (2019).

32. A. Vastermark, M. H. Saier, Jr., Time to Stop Holding the Elevator: A New Piece of the Transport Protein Mechanism Puzzle. *Structure (London, England : 1993)* **24**, 845-846 (2016).

33. B. H. Thurtle-Schmidt, R. M. Stroud, Structure of Bor1 supports an elevator transport mechanism for SLC4 anion exchangers. *Proceedings of the National Academy of Sciences of the United States of America* **113**, 10542-10546 (2016).

34. P. Luo *et al.*, Inward-facing conformation of l-ascorbate transporter suggests an elevator mechanism. *Cell discovery* **4**, 35 (2018).

35. Z. Ren *et al.*, Structure of an EIIC sugar transporter trapped in an inward-facing conformation. *Proceedings of the National Academy of Sciences of the United States of America* **115**, 5962-5967 (2018).

36. C. C. Su *et al.*, Structure and function of Neisseria gonorrhoeae MtrF illuminates a class of antimetabolite efflux pumps. *Cell reports* **11**, 61-70 (2015).

37. M. Montoya, An express elevator for Na+/H+. *Nature structural & molecular biology* **20**, 1144 (2013).

38. C. Lee *et al.*, A two-domain elevator mechanism for sodium/proton antiport. *Nature* **501**, 573-577 (2013).

39. M. Landreh *et al.*, Integrating mass spectrometry with MD simulations reveals the role of lipids in Na(+)/H(+) antiporters. *Nature communications* **8**, 13993 (2017).

40. C. Hunte *et al.*, Structure of a Na+/H+ antiporter and insights into mechanism of action and regulation by pH. *Nature* **435**, 1197-1202 (2005).

41. C. Lee *et al.*, Crystal structure of the sodium-proton antiporter NhaA dimer and new mechanistic insights. *The Journal of general physiology* **144**, 529-544 (2014).

42. M. Appel, D. Hizlan, K. R. Vinothkumar, C. Ziegler, W. Kühlbrandt, Conformations of NhaA, the Na/H exchanger from Escherichia coli, in the pH-activated and ion-translocating states. *Journal of molecular biology* **386**, 351-365 (2009).

43. C. Paulino, D. Wöhlert, E. Kapotova, Ö. Yildiz, W. Kühlbrandt, Structure and transport mechanism of the sodium/proton antiporter MjNhaP1. *eLife* **3**, e03583 (2014).

44. D. Wöhlert, W. Kühlbrandt, O. Yildiz, Structure and substrate ion binding in the sodium/proton antiporter PaNhaP. *eLife* **3**, e03579 (2014).

45. M. Coincon *et al.*, Crystal structures reveal the molecular basis of ion translocation in sodium/proton antiporters. *Nature structural & molecular biology* **23**, 248-255 (2016).

46. Y. Zhou, J. H. Bushweller, Solution structure and elevator mechanism of the membrane electron transporter CcdA. *Nature structural & molecular biology* **25**, 163-169 (2018).

47. E. R. Geertsma *et al.*, Structure of a prokaryotic fumarate transporter reveals the architecture of the SLC26 family. *Nature structural & molecular biology* **22**, 803-808 (2015).

48. P. Luo *et al.*, Crystal structure of a phosphorylation-coupled vitamin C transporter. *Nature structural & molecular biology* **22**, 238-241 (2015).

49. B. Byrne, It takes two to transport via an elevator. *Cell research* **27**, 965-966 (2017).

50. C. Mulligan *et al.*, The bacterial dicarboxylate transporter VcINDY uses a two-domain elevator-type mechanism. *Nature structural & molecular biology* **23**, 256-263 (2016).

51. J. R. Bolla *et al.*, Crystal structure of the Alcanivorax borkumensis YdaH transporter reveals an unusual topology. *Nature communications* **6**, 6874 (2015).

52. L. J. Swier, A. Guskov, D. J. Slotboom, Structural insight in the toppling mechanism of an energy-coupling factor transporter. *Nature communications* **7**, 11072 (2016).

53. P. Zhang, J. Wang, Y. Shi, Structure and mechanism of the S component of a bacterial ECF transporter. *Nature* **468**, 717-720 (2010).

54. G. B. Erkens *et al.*, The structural basis of modularity in ECF-type ABC transporters. *Nature structural & molecular biology* **18**, 755-760 (2011).

55. R. P. Berntsson *et al.*, Structural divergence of paralogous S components from ECF-type ABC transporters. *Proceedings of the National Academy of Sciences of the United States of America* **109**, 13990-13995 (2012).

56. N. K. Karpowich, J. Song, D. N. Wang, An Aromatic Cap Seals the Substrate Binding Site in an ECF-Type S Subunit for Riboflavin. *Journal of molecular biology* **428**, 3118-3130 (2016).

57. L. J. Swier *et al.*, Structure-based design of potent small-molecule binders to the S-component of the ECF transporter for thiamine. *Chembiochem : a European journal of chemical biology* **16**, 819-826 (2015).

58. Q. Zhao *et al.*, Structures of FolT in substrate-bound and substrate-released conformations reveal a gating mechanism for ECF transporters. *Nature communications* **6**, 7661 (2015).

59. Y. Alguel *et al.*, Structure of eukaryotic purine/H(+) symporter UapA suggests a role for homodimerization in transport activity. *Nature communications* **7**, 11336 (2016).
